# Montane diversification as a mechanism of speciation in neotropical butterflies

**DOI:** 10.1002/ece3.11704

**Published:** 2024-07-11

**Authors:** Luiza de Moraes Magaldi, Patrícia Eyng Gueratto, Enrique Ortega‐Abboud, Thadeu Sobral‐Souza, Mathieu Joron, Anete Pereira de Souza, André Victor Lucci Freitas, Karina Lucas Silva‐Brandão

**Affiliations:** ^1^ Departamento de Biologia Animal, Instituto de Biologia Universidade Estadual de Campinas Campinas SP Brazil; ^2^ Centre d'Ecologie Fonctionnelle et Evolutive, CNRS, EPHE, IRD Université de Montpellier Montpellier France; ^3^ Instituto de Biociências Universidade Federal de Mato Grosso Cuiabá MT Brazil; ^4^ Departamento de Biologia Vegetal, Instituto de Biologia Universidade Estadual de Campinas Campinas SP Brazil; ^5^ Leibniz Institute for the Analysis of Biodiversity Change Museum of Nature Hamburg Zoology Hamburg Germany

**Keywords:** *Actinote*, Atlantic forest, Nymphalidae, population genomics, speciation

## Abstract

The mountains in the Atlantic Forest domain are environments that harbor a high biodiversity, including species adapted to colder climates that were probably influenced by the climatic variations of the Pleistocene. To understand the phylogeographic pattern and assess the taxonomic boundaries between two sister montane species, a genomic study of the butterflies *Actinote mantiqueira* and *A. alalia* (Nymphalidae: Acraeini) was conducted. Analyses based on partial sequences of the mitochondrial gene COI (barcode region) failed to recover any phylogenetic or genetic structure discriminating the two species or sampling localities. However, single nucleotide polymorphisms gathered using Genotyping‐by‐Sequencing provided a strong isolation pattern in all analyses (genetic distance, phylogenetic hypothesis, clustering analyses, and *F*_ST_ statistics) which is consistent with morphology, separating all individuals of *A. alalia* from all populations of *A. mantiqueira*. The three sampled mountain ranges where *A. mantiqueira* populations occur—Serra do Mar, Serra da Mantiqueira, and Poços de Caldas Plateau—were identified as three isolated clusters. Paleoclimate simulations indicate that both species' distributions changed according to climatic oscillations in the Pleistocene period, with the two species potentially occurring in areas of lower altitude during glacial periods when compared to the interglacial periods (as the present). Besides, a potential path between their distribution through the Serra do Mar Mountain range was inferred. Therefore, the Pleistocene climatic fluctuation had a significant impact on the speciation process between *A. alalia* and *A. mantiqueira*, which was brought on by isolation at different mountain summits during interglacial periods, as shown by the modeled historical distribution and the observed genetic structure.

## Graphical abstract

We investigated taxonomic boundaries between two sister montane species using genomic data. SNPs gathered using GBS indicated a strong isolation pattern consistent with morphology, separating all individuals of *Actinote alalia* from all populations of *A. mantiqueira*. Pleistocene climatic fluctuation had a significant impact on the speciation process between the two species, which was brought on by isolation at different mountain summits during interglacial periods.

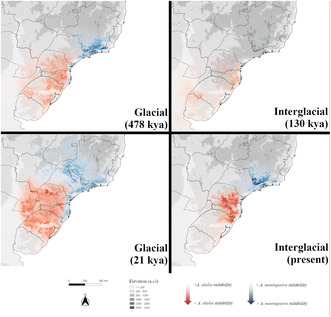

## 1 INTRODUCTION

Mountain environments are biodiversity hotspots that account for a third of all terrestrial species worldwide (Kohler et al., 2010; Körner et al., 2017). These environments resemble oceanic islands in various aspects, including their limited size, distinct boundaries, isolation, and dispersal restrictions. Due to these features, mountain ranges are often called “sky islands” (Gehrke & Linder, 2009; Hughes & Atchison, 2015; Sklenář et al., 2014).

Several intrinsic abiotic factors contribute to generating and maintaining high biodiversity in montane environments, such as topographic variation, heterogeneity of soil types, altitudinal gradients, and climatic variability (Antonelli et al., 2018; Badgley et al., 2017; Contreras‐Medina et al., 2003; Fischer et al., 2011; Fjeldså et al., 2012; Körner, 2004; Luebert & Muller, 2015). In general, two main mechanisms were proposed to explain the high diversification in montane areas: (1) the historical fragmentation of previously continuous habitats and dispersal associated with a rough landscape; and (2) the climate fluctuations over geologic time (Badgley, 2010; Knowles & Massatti, 2017; Mayr & Diamond, 1976; Patton & Smith, 1992). These mechanisms may trigger allopatric or parapatric speciation events in montane systems (Moritz et al., 2000; Vuilleumier & Monasterio, 1986), and for instance, several mechanisms of speciation in montane regions were proposed with variable resultant phylogenetic relationships patterns between sister taxa (Willmott et al., 2001).

Pleistocene climatic oscillations are well known for shaping the genetic diversity and geographical distribution of montane taxa (Colinvaux et al., 1997; do Amaral et al., 2021; Graham et al., 2014; Hewitt, 2004; Hooghiemstra & Van Der Hammen, 2004; Janzen, 1967; Mutke et al., 2014; Oswald & Steadman, 2015; Pie et al., 2018). Montane species responded to these climate fluctuations by shifting their distributions upward in periods of warm climate (Chen et al., 2009; Flantua et al., 2019; Freeman et al., 2018; Moritz et al., 2008). Accordingly, cold‐adapted species often remain isolated on mountain tops during warmer interglacial periods, undergoing genetic drift and divergence induced by natural selection (Brown, 1971; Fjeldså, 1994; Flantua et al., 2019; Ramírez‐Barahona & Eguiarte, 2013; Safford, 1999). On the other hand, species range can extend to lower areas during colder periods, connecting previously separate locales, perhaps leading to the mixing of populations and the formation of hybrids (Donoghue et al., 2014; Petit et al., 2003). In the last case, populations can even expand into previously unsuitable regions, triggering diversification through dispersal and settlement in new areas (“dispersification”; Moore & Donoghue, 2007).

In general, species are expected to either move to follow favorable conditions (i.e., range shifts, both in latitude and elevation, or contractions) or persist in the landscape through evolution to novel environmental conditions (e.g., phenotypic plasticity or adaptation) in response to climatic change (Costello et al., 2022; Waldvogel et al., 2020). Currently, most of the knowledge on montane environments has focused on vertebrates or vascular plants (Wang et al., 2024). Notwithstanding, changes due to climatic change were described for European alpine burnet moth species in the Pyrenees (Dieker et al., 2011). In this sense, invertebrate montane species are especially concerning due to the high degree of specialization that montane species often exhibit within narrow temperature bands. The upslope movements are predicted to result in a reduction in their potential area of occupancy and become more vulnerable to the stochastic extinctions that characterize small populations (Elsen & Tingley, 2015).

The Neotropical region shelters the Andes, the longest mountain range on Earth. It harbors a rich biota, which has been investigated as a paradigm for research on the patterns and processes of montane diversification (Adams, 1985; Bacon et al., 2018; Cadena, 2007; Castroviejo‐Fisher et al., 2014; Chazot et al., 2016; Elias et al., 2009; Hall, 2005; Hazzi et al., 2018; Kessler, 2001; Pyrcz & Wojtusiak, 2002; Viloria, 2003; Willmott et al., 2001). In the Atlantic Forest, a highly threatened biome, there are four massifs: the Serra do Mar, the Serra da Mantiqueira, the Serra Geral and the Espinhaço mountain ranges, ranging from sea level to 2891 m (Moreira & Camelier, 1977). These mountains are located between 15° and 30° South and are mostly covered by tropical or subtropical forests, with climatic variations ranging from no dry season near the coast (ombrophilous dense forests) to the presence of a marked dry season in the interior (semi‐deciduous forests with physiological drought and mean temperature below 15°C) (Morellato & Haddad, 2000; Oliveira‐Filho & Fontes, 2000; Veloso et al., 1991). During the Late Pleistocene, an expansion of high‐altitude grasslands took place in the Montane Atlantic Forest (hereafter MAF), reflecting the colder and drier conditions that became predominant afterward (Behling, 2002; Behling et al., 2007; Behling & Safford, 2010).

The phylogeography of the Atlantic Forest is mainly known for vertebrates and plants with low or wide elevational ranges, and points to a clear latitudinal split that isolates the northern and southern diversity, primarily in the central corridor (Peres et al., 2020). However, no common pattern was found for high‐altitude species of the MAF (Amaro et al., 2012; Batalha‐Filho et al., 2012; Firkowski et al., 2016; Françoso et al., 2016; Peres et al., 2015; Thom et al., 2020). This apparent lack of a common pattern may be the result of various processes that have diverse effects on taxa. For instance, Pleistocene climate oscillations may have shaped the genetic diversity of montane populations for certain taxa within the MAF, such as bees and birds (do Amaral et al., 2021; Françoso et al., 2016; Thom et al., 2020). On the other hand, stability in population size during the Pleistocene was observed for other taxa, such as spiders, frogs, and birds (Amaro et al., 2012; Batalha‐Filho et al., 2012; Peres et al., 2015). Additionally, the population structure of some MAF species show phylogeographic splits that correspond to divergence across river barriers (Amaro et al., 2012; Françoso et al., 2016). Others, however, show a spectrum of phenotypic and genetic divergence across an inter‐mountain valley, defined as the São Paulo subtropical gap, around 20°S (do Amaral et al., 2021; Thom et al., 2020). Still, endemic populations showing no genetic structure were observed as well (Batalha‐Filho et al., 2012; Françoso et al., 2016; Mota et al., 2020; Peres et al., 2015). Thus, new phylogeographic research on invertebrates can contribute to revealing the processes of diversification that have culminated in this variety of patterns observed for highland species (Peres et al., 2015).

For instance, numerous butterfly species exhibit a restricted or favored distribution at higher elevations, despite the scarcity of knowledge regarding their evolutionary divergence within the MAF (Wang et al., 2024). Butterflies are well‐known model organisms for various ecological and evolutionary studies and are especially rich and diversified in the Atlantic Forest, including many endemic taxa (Brown Jr & Freitas, 2000; Santos et al., 2018; Watt & Boggs, 2003). A few studies focusing on the distribution of butterfly diversity in the Atlantic Forest point to a North‐South pattern of diversification, following the two bioclimatic regions of the biome (Brown et al., 2020; Pablos et al., 2021; Paz et al., 2021; Seraphim et al., 2016), while virtually nothing is known about the pattern of divergence for montane species.

The nymphalid butterflies of the genus *Actinote* Hübner [1819] (Heliconiinae: Acraeini) include 38 described species, the vast majority of which are found in the highlands of the Atlantic Forest in southeast Brazil (Freitas et al., 2018, 2020; Gueratto, 2023; Lamas, 2004; Paluch, 2006; Paluch & Casagrande, 2006). In the MAF, there are seven known species of *Actinote* that participate in a Müllerian mimicry ring called “orangish‐red mimicry complex” (Francini, 1989; Freitas et al., 2018). The species in this complex are characterized by a dark orange and brown dorsal striped pattern, with a ventral design that is somewhat variable among species. The butterflies of the orangish‐red mimicry complex, including *Actinote alalia* (Felder & Felder, 1860) and *A. mantiqueira* Freitas et al., 2018, are remarkably morphologically similar and difficult to distinguish due to the strong similarities in wing pattern, mainly concerning female wing color pattern, and high intraspecific variation (D'Almeida, 1935; Francini, 1989; Francini & Penz, 2006; Freitas et al., 2018; Paluch & Casagrande, 2006). *Actinote mantiqueira* is a recently described species distributed in the Serra do Mar and Serra da Mantiqueira, at altitudes from 1000 to 2000 m, in sites usually characterized by a well‐preserved montane ombrophilous forest (Freitas et al., 2018). The species is geographically separated from its sister species, *A. alalia*, which occurs in the montane areas of the southernmost Brazil, at altitudes from 800 to 1400 m, associated with preserved montane forest. Adults of both sexes of *A. alalia* are easily observed in forest edges and areas of contact between forest and high‐altitude grasslands (Freitas et al., 2018).

**FIGURE 1:**
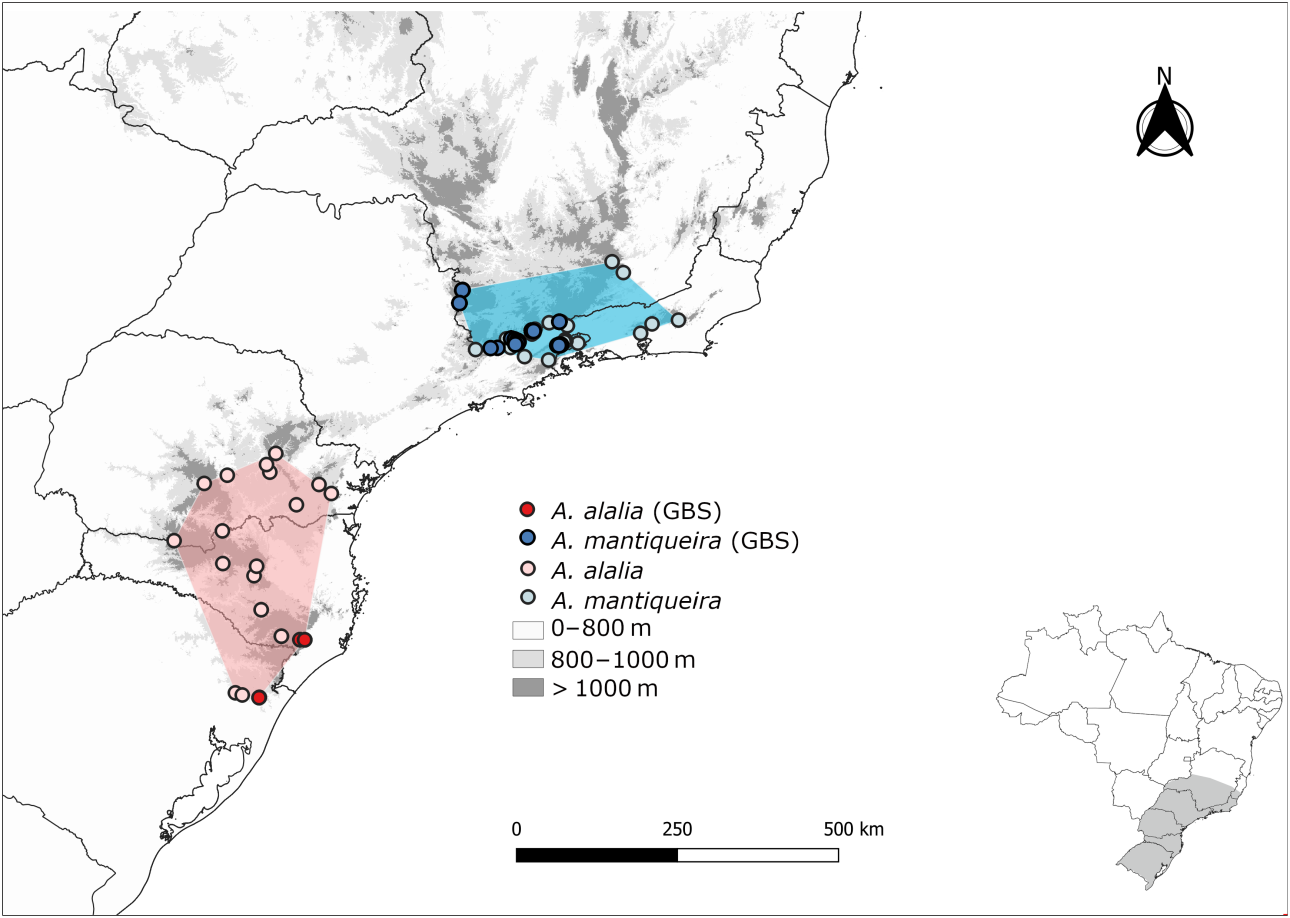
Map of the sampling locations for *Actinote mantiqueira* (blue circles) and *A. alalia* (red circles). Shaded polygons and lighter circles represent the known distribution and localities of occurrence for each species.

The current geographic distribution of these sister species allows us to test the phylogeographic hypotheses proposed for the MAF species since they are related to distinct paleoclimatic regions of the Atlantic Forest (defined by Ledru et al. (2017)) and are distributed in distinct mountain blocks: *A. mantiqueira* occurs in the Central Atlantic Forest (CAF), between 15° and 23°S, whereas *A. alalia* has small populations restrict to a few sites throughout the South Atlantic Forest (SAF), from 23° to 30°S (Figure 1). The distribution pattern of these sister species (Silva‐Brandão et al., 2008) is consistent with the hypothesis that historical climatic changes played a significant role in shaping their current range. In this particular case, this gap between their geographic distribution is a natural occurrence and not due to habitat loss, as the forests are continuous along the coastal mountains within the range of both species (Freitas et al., 2018; Morellato & Haddad, 2000). The isolation of populations on different mountain ranges due to temperature increases likely contributed to the allopatric speciation observed between montane regions (Willmott et al., 2001).

To test this hypothesis, a phylogeographic study of the pair of sister species, *A. mantiqueira* and *A. alalia*, was conducted to infer their genetic variability and population structure. First, the species limit between these two species was tested using two molecular markers, the COI barcode region (Hebert et al., 2003), which is widely investigated to infer butterflies' taxonomy (Silva‐Brandão et al., 2009), and single nucleotide polymorphisms (SNPs) obtained with the genotyping‐by‐sequencing technique (GBS) (Elshire et al., 2011; Poland et al., 2012). Additionally, the ecological niche of each species was modeled from the present to 800 thousand years ago (kya), including nine glacial‐interglacial cycles, to estimate the influence of past climate variation on their distribution and test whether their niche could have expanded under colder conditions. These two species are found predominantly on the top of the Atlantic Forest mountains. Therefore, they represent a high conservation priority globally since mountaintop species stand to face local extinction with upslope range shifts regardless of underlying topography. Using genomic markers, we were able to evaluate the effect of the present disjunct distribution on different mountain ranges in the population genomic structure in both species. Based on the morphological differences of *A. mantiqueira* and *A. alalia* and on the results reported for other species of the MAF that present similar distributions, a well‐defined genetic structure between these two species is expected, which would also indicate that the São Paulo subtropical gap also functions as geographic barrier for MAF butterflies (do Amaral et al., 2021; Freitas et al., 2018; Thom et al., 2020).

## 2 MATERIALS AND METHODS

### 2.1 Sampling and gDNA extraction

A total of 144 individuals were sampled from 17 localities, comprising eight specimens of *A. alalia* (the low *N* is due to its rarity at the visited sites) and 136 of *A. mantiqueira* (Figure 1, Table 1). Three individuals corresponding to *A. catarina* and two samples of *A. dalmeidai* were also collected from the same localities to be used as outgroups in some analyses (names in bold in Table 1). The localities (municipalities) were defined as populations for downstream analyses. Ten localities were sampled for *A. mantiqueira*, representing most of its known distribution. For *A. alalia*, however, fresh samples were obtained from two localities throughout its distribution.

**TABLE 1:** Voucher collection data: Species codes, collection locality, mountain range, marker used, and the Genbank accession number for the mitochondrial sequences. Names of species in bold refer to specimens used as outgroups in some analyses.

Voucher	Species	Locality	Population code	Mountain Range	Marker	Genbank accession number
BLU‐0377	***Actinote catarina***	FLONA São Francisco de Paula ‐ RS		Serra Geral	Barcode	PP885781
ALA BJ2/ ac230	***Actinote dalmeidai***	Bom Jardim da Serra ‐ SC		Serra Geral	GBS	
ALA I2‐7	***Actinote dalmeidai***	Pq. Nac. Itatiaia		Serra da Mantiqueira	GBS	
ALA CF‐1	*Actinote alalia*	Cânion do Funil, Bom Jardim da Serra ‐ SC	aBJ	Serra Geral	GBS	
ALA CF‐2	*Actinote alalia*	Cânion do Funil, Bom Jardim da Serra ‐ SC	aBJ	Serra Geral	GBS	
ALA CF‐3	*Actinote alalia*	Cânion do Funil, Bom Jardim da Serra ‐ SC	aBJ	Serra Geral	GBS/ Barcode	PP885751
ALA CF‐4	*Actinote alalia*	Cânion do Funil, Bom Jardim da Serra ‐ SC	aBJ	Serra Geral	GBS/ Barcode	PP885752
ALA CF‐5	*Actinote alalia*	Cânion do Funil, Bom Jardim da Serra ‐ SC	aBJ	Serra Geral	GBS	
ALA CF‐6	*Actinote alalia*	Cânion do Funil, Bom Jardim da Serra ‐ SC	aBJ	Serra Geral	GBS	
ALA BJ1/ ac228	*Actinote alalia*	Bom Jardim da Serra ‐ SC	aBJ	Serra Geral	GBS/ Barcode	PP885742
ac229	*Actinote alalia*	Bom Jardim da Serra ‐ SC		Serra Geral	Barcode	PP885743
ALA FL1	*Actinote alalia*	FLONA São Francisco de Paula ‐ RS	aFL	Serra Geral	GBS	
ALA FL2	*Actinote alalia*	FLONA São Francisco de Paula ‐ RS	aFL	Serra Geral	GBS/ Barcode	PP885754
ac181	*Actinote alalia*	FLONA São Francisco de Paula ‐ RS		Serra Geral	Barcode	PP885741
ac231	*Actinote alalia*	FLONA São Francisco de Paula ‐ RS		Serra Geral	Barcode	PP885744
ac90	*Actinote alalia*	FLONA São Francisco de Paula ‐ RS		Serra Geral	Barcode	EU275618
ac148	*Actinote alalia*	FLONA São Francisco de Paula ‐ RS		Serra Geral	Barcode	PP885736
ac149	*Actinote alalia*	FLONA São Francisco de Paula ‐ RS		Serra Geral	Barcode	PP885737
ac117	*Actinote alalia*	FLONA São Francisco de Paula ‐ RS		Serra Geral	Barcode	PP885735
ac77	*Actinote alalia*	Curitiba ‐ PR		Serra do Mar (South)	Barcode	EU275617
ALA B2 ‐ 1	*Actinote mantiqueira*	Serra da Bocaina, Cunha ‐ SP	mBoc	Serra do Mar (Bocaina)	GBS	
ALA B2 ‐ 2	*Actinote mantiqueira*	Serra da Bocaina, Cunha ‐ SP	mBoc	Serra do Mar (Bocaina)	GBS	
ALA B2 ‐ 3	*Actinote mantiqueira*	Serra da Bocaina, Cunha ‐ SP	mBoc	Serra do Mar (Bocaina)	GBS	
ALA B2 ‐ 4	*Actinote mantiqueira*	Serra da Bocaina, Cunha ‐ SP	mBoc	Serra do Mar (Bocaina)	GBS	
ALA B2 ‐ 5	*Actinote mantiqueira*	Serra da Bocaina, Cunha ‐ SP	mBoc	Serra do Mar (Bocaina)	GBS	
ALA B3 ‐ 1	*Actinote mantiqueira*	Serra da Bocaina, Cunha ‐ SP	mBoc	Serra do Mar (Bocaina)	GBS/ Barcode	PP885745
ALA B4 ‐ 1	*Actinote mantiqueira*	Serra da Bocaina, Cunha ‐ SP	mBoc	Serra do Mar (Bocaina)	GBS	
ALA B4 ‐ 2	*Actinote mantiqueira*	Serra da Bocaina, Cunha ‐ SP	mBoc	Serra do Mar (Bocaina)	GBS	
ALA B4 ‐ 3	*Actinote mantiqueira*	Serra da Bocaina, Cunha ‐ SP	mBoc	Serra do Mar (Bocaina)	GBS	
ALA B5‐1	*Actinote mantiqueira*	Serra da Bocaina, Cunha ‐ SP	mBoc	Serra do Mar (Bocaina)	GBS	
ALA B5‐2	*Actinote mantiqueira*	Serra da Bocaina, Cunha ‐ SP	mBoc	Serra do Mar (Bocaina)	GBS	
ALA B5‐3	*Actinote mantiqueira*	Serra da Bocaina, Cunha ‐ SP	mBoc	Serra do Mar (Bocaina)	GBS/ Barcode	PP885746
ALA B5‐4	*Actinote mantiqueira*	Serra da Bocaina, Cunha ‐ SP	mBoc	Serra do Mar (Bocaina)	GBS	
ALA B5‐5	*Actinote mantiqueira*	Serra da Bocaina, Cunha ‐ SP	mBoc	Serra do Mar (Bocaina)	GBS	
ALA B5‐6	*Actinote mantiqueira*	Serra da Bocaina, Cunha ‐ SP	mBoc	Serra do Mar (Bocaina)	GBS	
ALA BH1‐1	*Actinote mantiqueira*	Serra da Bocaina, Cunha ‐ SP	mBoc	Serra do Mar (Bocaina)	GBS	
ALA BH1‐2	*Actinote mantiqueira*	Serra da Bocaina, Cunha ‐ SP	mBoc	Serra do Mar (Bocaina)	GBS/ Barcode	PP885747
ALA BH1‐3	*Actinote mantiqueira*	Serra da Bocaina, Cunha ‐ SP	mBoc	Serra do Mar (Bocaina)	GBS/ Barcode	PP885748
ALA BH1‐4	*Actinote mantiqueira*	Serra da Bocaina, Cunha ‐ SP	mBoc	Serra do Mar (Bocaina)	GBS/ Barcode	PP885749
ALA BH1‐5	*Actinote mantiqueira*	Serra da Bocaina, Cunha ‐ SP	mBoc	Serra do Mar (Bocaina)	GBS	
ALA BH2‐1	*Actinote mantiqueira*	Serra da Bocaina, Cunha ‐ SP	mBoc	Serra do Mar (Bocaina)	GBS	
ALA BH2‐2	*Actinote mantiqueira*	Serra da Bocaina, Cunha ‐ SP	mBoc	Serra do Mar (Bocaina)	GBS	
ALA BH2‐3	*Actinote mantiqueira*	Serra da Bocaina, Cunha ‐ SP	mBoc	Serra do Mar (Bocaina)	GBS	
ALA BH2‐4	*Actinote mantiqueira*	Serra da Bocaina, Cunha ‐ SP	mBoc	Serra do Mar (Bocaina)	GBS	
ALA BM‐1	*Actinote mantiqueira*	Serra da Bocaina, Cunha ‐ SP	mBoc	Serra do Mar (Bocaina)	GBS	
ALA BI‐2	*Actinote mantiqueira*	Serra da Bocaina, Cunha ‐ SP		Serra do Mar (Bocaina)	Barcode	PP885750
ac176	*Actinote mantiqueira*	Serra da Bocaina, Silveiras ‐ SP		Serra do Mar (Bocaina)	Barcode	PP885738
ac179	*Actinote mantiqueira*	Serra da Bocaina, Silveiras ‐ SP		Serra do Mar (Bocaina)	Barcode	PP885739
ac180	*Actinote mantiqueira*	Serra da Bocaina, Silveiras ‐ SP		Serra do Mar (Bocaina)	Barcode	PP885740
ALA IM1	*Actinote mantiqueira*	Pico do Imbiri, Campos do Jordão ‐ SP	mCJ	Serra da Mantiqueira	GBS	
ALA IM2	*Actinote mantiqueira*	Pico do Imbiri, Campos do Jordão ‐ SP	mCJ	Serra da Mantiqueira	GBS	
ALA IM3	*Actinote mantiqueira*	Pico do Imbiri, Campos do Jordão ‐ SP	mCJ	Serra da Mantiqueira	GBS	
ALA IM4	*Actinote mantiqueira*	Pico do Imbiri, Campos do Jordão ‐ SP	mCJ	Serra da Mantiqueira	GBS/ Barcode	PP885759
ALA IM5	*Actinote mantiqueira*	Pico do Imbiri, Campos do Jordão ‐ SP	mCJ	Serra da Mantiqueira	GBS	
ALA MF‐1	*Actinote mantiqueira*	Mirante Ferradura, Campos do Jordão ‐ SP	mCJ	Serra da Mantiqueira	GBS	
ALA MF‐2	*Actinote mantiqueira*	Mirante Ferradura, Campos do Jordão ‐ SP	mCJ	Serra da Mantiqueira	GBS	
ALA MF‐3	*Actinote mantiqueira*	Mirante Ferradura, Campos do Jordão ‐ SP	mCJ	Serra da Mantiqueira	GBS	
ALA MF‐4	*Actinote mantiqueira*	Mirante Ferradura, Campos do Jordão ‐ SP	mCJ	Serra da Mantiqueira	GBS	
ALA MF‐5	*Actinote mantiqueira*	Mirante Ferradura, Campos do Jordão ‐ SP	mCJ	Serra da Mantiqueira	GBS	
ALA NC‐6	*Actinote mantiqueira*	Novo Capivari, Campos do Jordão ‐ SP	mCJ	Serra da Mantiqueira	GBS	
ALA NC‐1	*Actinote mantiqueira*	Novo Capivari, Campos do Jordão ‐ SP	mCJ	Serra da Mantiqueira	GBS	
ALA NC‐3	*Actinote mantiqueira*	Novo Capivari, Campos do Jordão ‐ SP	mCJ	Serra da Mantiqueira	GBS/ Barcode	PP885761
ALA NC‐4	*Actinote mantiqueira*	Novo Capivari, Campos do Jordão ‐ SP	mCJ	Serra da Mantiqueira	GBS/ Barcode	PP885762
ALA NC‐5	*Actinote mantiqueira*	Novo Capivari, Campos do Jordão ‐ SP	mCJ	Serra da Mantiqueira	GBS	
ALA NC2‐2	*Actinote mantiqueira*	Novo Capivari, Campos do Jordão ‐ SP	mCJ	Serra da Mantiqueira	GBS	
ALA NC2‐3	*Actinote mantiqueira*	Novo Capivari, Campos do Jordão ‐ SP	mCJ	Serra da Mantiqueira	GBS	
ALA NC2‐4	*Actinote mantiqueira*	Novo Capivari, Campos do Jordão ‐ SP	mCJ	Serra da Mantiqueira	GBS	
ALA NC2‐5	*Actinote mantiqueira*	Novo Capivari, Campos do Jordão ‐ SP	mCJ	Serra da Mantiqueira	GBS	
ALA NC2‐6	*Actinote mantiqueira*	Novo Capivari, Campos do Jordão ‐ SP	mCJ	Serra da Mantiqueira	GBS/ Barcode	PP885760
ALA PI‐1	*Actinote mantiqueira*	Est. Pico do Itapeva, Campos do Jordão ‐ SP	mCJ	Serra da Mantiqueira	GBS	
ALA PI3‐1	*Actinote mantiqueira*	Est. Pico do Itapeva, Campos do Jordão ‐ SP	mCJ	Serra da Mantiqueira	GBS	
ALA PI3‐2	*Actinote mantiqueira*	Est. Pico do Itapeva, Campos do Jordão ‐ SP	mCJ	Serra da Mantiqueira	GBS	
ALA PI3‐3	*Actinote mantiqueira*	Est. Pico do Itapeva, Campos do Jordão ‐ SP	mCJ	Serra da Mantiqueira	GBS/ Barcode	PP885778
ALA PI3‐4	*Actinote mantiqueira*	Est. Pico do Itapeva, Campos do Jordão ‐ SP	mCJ	Serra da Mantiqueira	GBS/ Barcode	PP885779
ALA PI3‐5	*Actinote mantiqueira*	Est. Pico do Itapeva, Campos do Jordão ‐ SP	mCJ	Serra da Mantiqueira	GBS/ Barcode	PP885780
BLU‐1026	*Actinote mantiqueira*	Pico do Imbiri, Campos do Jordão ‐ SP		Serra da Mantiqueira	Barcode	PP885786
BLU‐0787	*Actinote mantiqueira*	Campos do Jordão ‐ SP		Serra da Mantiqueira	Barcode	PP885782
BLU‐0791	*Actinote mantiqueira*	Campos do Jordão ‐ SP		Serra da Mantiqueira	Barcode	PP885783
BLU‐0799	*Actinote mantiqueira*	Campos do Jordão ‐ SP		Serra da Mantiqueira	Barcode	PP885784
ac9	*Actinote mantiqueira*	Campos do Jordão ‐ SP		Serra da Mantiqueira	Barcode	EU275574
ac113	*Actinote mantiqueira*	Campos do Jordão ‐ SP		Serra da Mantiqueira	Barcode	PP885734
ALA D2‐1	*Actinote mantiqueira*	Delfim Moreira ‐ MG	mDM	Serra da Mantiqueira	GBS	
ALA D4‐1	*Actinote mantiqueira*	Delfim Moreira ‐ MG	mDM	Serra da Mantiqueira	GBS	
ALA D4‐2	*Actinote mantiqueira*	Delfim Moreira ‐ MG	mDM	Serra da Mantiqueira	GBS/ Barcode	PP885753
ALA D4‐3	*Actinote mantiqueira*	Delfim Moreira ‐ MG	mDM	Serra da Mantiqueira	GBS	
ALA D4‐4	*Actinote mantiqueira*	Delfim Moreira ‐ MG	mDM	Serra da Mantiqueira	GBS	
ALA D4‐5	*Actinote mantiqueira*	Delfim Moreira ‐ MG	mDM	Serra da Mantiqueira	GBS	
ALA D5‐1	*Actinote mantiqueira*	Delfim Moreira ‐ MG	mDM	Serra da Mantiqueira	GBS	
ALA D5‐2	*Actinote mantiqueira*	Delfim Moreira ‐ MG	mDM	Serra da Mantiqueira	GBS	
ALA D5‐3	*Actinote mantiqueira*	Delfim Moreira ‐ MG	mDM	Serra da Mantiqueira	GBS	
ALA D6 ‐1	*Actinote mantiqueira*	Delfim Moreira ‐ MG	mDM	Serra da Mantiqueira	GBS	
ALA D6 ‐2	*Actinote mantiqueira*	Delfim Moreira ‐ MG	mDM	Serra da Mantiqueira	GBS	
BLU‐1040	*Actinote mantiqueira*	Delfim Moreira ‐ MG	mDM	Serra da Mantiqueira	GBS/ Barcode	PP885787
BLU‐1041	*Actinote mantiqueira*	Delfim Moreira ‐ MG	mDM	Serra da Mantiqueira	GBS/ Barcode	PP885788
BLU‐1042	*Actinote mantiqueira*	Delfim Moreira ‐ MG	mDM	Serra da Mantiqueira	GBS/ Barcode	PP885789
ALA PM ‐ 1	*Actinote mantiqueira*	Pico Marins, Delfim Moreira ‐ MG	mDM	Serra da Mantiqueira	GBS	
ALA PM ‐ 2	*Actinote mantiqueira*	Pico Marins, Delfim Moreira ‐ MG	mDM	Serra da Mantiqueira	GBS	
ALA PM ‐ 3	*Actinote mantiqueira*	Pico Marins, Delfim Moreira ‐ MG	mDM	Serra da Mantiqueira	GBS	
ALA PM ‐ 4	*Actinote mantiqueira*	Pico Marins, Delfim Moreira ‐ MG	mDM	Serra da Mantiqueira	GBS	
ALA PM ‐ 5	*Actinote mantiqueira*	Pico Marins, Delfim Moreira ‐ MG	mDM	Serra da Mantiqueira	GBS	
ALA FX1	*Actinote mantiqueira*	Pouso do Rochedo, S. Fransisco Xavier ‐ SP	mFX	Serra da Mantiqueira	GBS	
BLU‐0878	*Actinote mantiqueira*	Pouso do Rochedo, Alto São Francisco de Xavier ‐ SP		Serra da Mantiqueira	Barcode	PP885785
ALA I2‐1	*Actinote mantiqueira*	Pq. Nac. Itatiaia	mI	Serra da Mantiqueira	GBS	
ALA I2‐10	*Actinote mantiqueira*	Pq. Nac. Itatiaia	mI	Serra da Mantiqueira	GBS	
ALA I2‐11	*Actinote mantiqueira*	Pq. Nac. Itatiaia	mI	Serra da Mantiqueira	GBS/ Barcode	PP885756
ALA I2‐12	*Actinote mantiqueira*	Pq. Nac. Itatiaia	mI	Serra da Mantiqueira	GBS	
ALA I2‐13	*Actinote mantiqueira*	Pq. Nac. Itatiaia	mI	Serra da Mantiqueira	GBS/ Barcode	PP885757
ALA I2‐14	*Actinote mantiqueira*	Pq. Nac. Itatiaia	mI	Serra da Mantiqueira	GBS	
ALA I2‐15	*Actinote mantiqueira*	Pq. Nac. Itatiaia	mI	Serra da Mantiqueira	GBS	
ALA I2‐2	*Actinote mantiqueira*	Pq. Nac. Itatiaia	mI	Serra da Mantiqueira	GBS	
ALA I2‐4	*Actinote mantiqueira*	Pq. Nac. Itatiaia	mI	Serra da Mantiqueira	GBS	
ALA I2‐5	*Actinote mantiqueira*	Pq. Nac. Itatiaia	mI	Serra da Mantiqueira	GBS/ Barcode	PP885758
ALA I2‐6	*Actinote mantiqueira*	Pq. Nac. Itatiaia	mI	Serra da Mantiqueira	GBS	
ALA I2‐8	*Actinote mantiqueira*	Pq. Nac. Itatiaia	mI	Serra da Mantiqueira	GBS	
ALA I2‐9	*Actinote mantiqueira*	Pq. Nac. Itatiaia	mI	Serra da Mantiqueira	GBS	
ALA I2	*Actinote mantiqueira*	Pq. Nac. Itatiaia	mI	Serra da Mantiqueira	Barcode	PP885755
ALA MV2‐1	*Actinote mantiqueira*	Av. Aeroporto, Monte Verde ‐ MG	mMV	Serra da Mantiqueira	GBS	
BLU‐1039	*Actinote mantiqueira*	Est. Pico do Diamante, Pindamonhangaba ‐ SP	mPD	Serra da Mantiqueira	GBS	
ALA PD‐3	*Actinote mantiqueira*	Est. Pico do Diamante, Pindamonhangaba ‐ SP	mPD	Serra da Mantiqueira	GBS	
ALA PD‐4	*Actinote mantiqueira*	Est. Pico do Diamante, Pindamonhangaba ‐ SP	mPD	Serra da Mantiqueira	GBS	
ALA PD‐5	*Actinote mantiqueira*	Est. Pico do Diamante, Pindamonhangaba ‐ SP	mPD	Serra da Mantiqueira	GBS	
ALA PD‐6	*Actinote mantiqueira*	Est. Pico do Diamante, Pindamonhangaba ‐ SP	mPD	Serra da Mantiqueira	GBS	
ac36	*Actinote mantiqueira*	Pico do Itapeva, Pindamonhangaba ‐ SP		Serra da Mantiqueira	Barcode	EU275575
ALA SB‐1	*Actinote mantiqueira*	Pedra do Baú, São Bento do Sapucaí ‐ SP	mSB	Serra da Mantiqueira	GBS	
ALA SB‐10	*Actinote mantiqueira*	Pedra do Baú, São Bento do Sapucaí ‐ SP	mSB	Serra da Mantiqueira	GBS	
ALA SB‐11	*Actinote mantiqueira*	Pedra do Baú, São Bento do Sapucaí ‐ SP	mSB	Serra da Mantiqueira	GBS	
ALA SB‐2	*Actinote mantiqueira*	Pedra do Baú, São Bento do Sapucaí ‐ SP	mSB	Serra da Mantiqueira	GBS	
ALA SB‐3	*Actinote mantiqueira*	Pedra do Baú, São Bento do Sapucaí ‐ SP	mSB	Serra da Mantiqueira	GBS	
ALA SB‐4	*Actinote mantiqueira*	Pedra do Baú, São Bento do Sapucaí ‐ SP	mSB	Serra da Mantiqueira	GBS	
ALA SB‐5	*Actinote mantiqueira*	Pedra do Baú, São Bento do Sapucaí ‐ SP	mSB	Serra da Mantiqueira	GBS	
ALA SB‐6	*Actinote mantiqueira*	Pedra do Baú, São Bento do Sapucaí ‐ SP	mSB	Serra da Mantiqueira	GBS	
ALA SB‐7	*Actinote mantiqueira*	Pedra do Baú, São Bento do Sapucaí ‐ SP	mSB	Serra da Mantiqueira	GBS	
ALA SB‐8	*Actinote mantiqueira*	Pedra do Baú, São Bento do Sapucaí ‐ SP	mSB	Serra da Mantiqueira	GBS	
ALA SB‐9	*Actinote mantiqueira*	Pedra do Baú, São Bento do Sapucaí ‐ SP	mSB	Serra da Mantiqueira	GBS	
ALA PC1‐01	*Actinote mantiqueira*	Cristo Redentor, Poços de Caldas ‐ MG	mPC	Poços de Caldas Plateau	GBS	
ALA PC1‐02	*Actinote mantiqueira*	Cristo Redentor, Poços de Caldas ‐ MG	mPC	Poços de Caldas Plateau	GBS/ Barcode	PP885763
ALA PC1‐03	*Actinote mantiqueira*	Cristo Redentor, Poços de Caldas ‐ MG	mPC	Poços de Caldas Plateau	GBS	
ALA PC1‐04	*Actinote mantiqueira*	Cristo Redentor, Poços de Caldas ‐ MG	mPC	Poços de Caldas Plateau	GBS/ Barcode	PP885764
ALA PC1‐05	*Actinote mantiqueira*	Cristo Redentor, Poços de Caldas ‐ MG	mPC	Poços de Caldas Plateau	GBS	
ALA PC1‐06	*Actinote mantiqueira*	Cristo Redentor, Poços de Caldas ‐ MG	mPC	Poços de Caldas Plateau	GBS/ Barcode	PP885765
ALA PC1‐07	*Actinote mantiqueira*	Cristo Redentor, Poços de Caldas ‐ MG	mPC	Poços de Caldas Plateau	GBS	
ALA PC1‐08	*Actinote mantiqueira*	Cristo Redentor, Poços de Caldas ‐ MG	mPC	Poços de Caldas Plateau	GBS/ Barcode	PP885766
ALA PC1‐09	*Actinote mantiqueira*	Cristo Redentor, Poços de Caldas ‐ MG	mPC	Poços de Caldas Plateau	GBS/ Barcode	PP885767
ALA PC1‐10	*Actinote mantiqueira*	Cristo Redentor, Poços de Caldas ‐ MG	mPC	Poços de Caldas Plateau	GBS	
ALA PC1‐11	*Actinote mantiqueira*	Cristo Redentor, Poços de Caldas ‐ MG	mPC	Poços de Caldas Plateau	GBS	
ALA PC1‐12	*Actinote mantiqueira*	Cristo Redentor, Poços de Caldas ‐ MG	mPC	Poços de Caldas Plateau	GBS	
ALA PC1‐13	*Actinote mantiqueira*	Cristo Redentor, Poços de Caldas ‐ MG	mPC	Poços de Caldas Plateau	GBS	
ALA PC1‐14	*Actinote mantiqueira*	Cristo Redentor, Poços de Caldas ‐ MG	mPC	Poços de Caldas Plateau	GBS	
ALA PC1‐15	*Actinote mantiqueira*	Cristo Redentor, Poços de Caldas ‐ MG	mPC	Poços de Caldas Plateau	GBS/ Barcode	PP885768
ALA PC1‐16	*Actinote mantiqueira*	Cristo Redentor, Poços de Caldas ‐ MG	mPC	Poços de Caldas Plateau	GBS/ Barcode	PP885769
ALA PC1‐17	*Actinote mantiqueira*	Cristo Redentor, Poços de Caldas ‐ MG	mPC	Poços de Caldas Plateau	GBS/ Barcode	PP885770
ALA PC1‐18	*Actinote mantiqueira*	Cristo Redentor, Poços de Caldas ‐ MG	mPC	Poços de Caldas Plateau	GBS	
ALA PC1‐19	*Actinote mantiqueira*	Cristo Redentor, Poços de Caldas ‐ MG	mPC	Poços de Caldas Plateau	GBS/ Barcode	PP885771
ALA PC1‐20	*Actinote mantiqueira*	Cristo Redentor, Poços de Caldas ‐ MG	mPC	Poços de Caldas Plateau	GBS/ Barcode	PP885772
ALA PC2‐1	*Actinote mantiqueira*	Poços de Caldas ‐ MG	mPC	Poços de Caldas Plateau	GBS	
ALA PC2‐2	*Actinote mantiqueira*	Poços de Caldas ‐ MG	mPC	Poços de Caldas Plateau	GBS	
ALA PG‐1	*Actinote mantiqueira*	Pico do Gavião, Andradas ‐ MG	mPG	Poços de Caldas Plateau	GBS	
ALA PG‐10	*Actinote mantiqueira*	Pico do Gavião, Andradas ‐ MG	mPG	Poços de Caldas Plateau	GBS/ Barcode	PP885775
ALA PG‐11	*Actinote mantiqueira*	Pico do Gavião, Andradas ‐ MG	mPG	Poços de Caldas Plateau	GBS/ Barcode	PP885776
ALA PG‐12	*Actinote mantiqueira*	Pico do Gavião, Andradas ‐ MG	mPG	Poços de Caldas Plateau	GBS/ Barcode	PP885777
ALA PG‐2	*Actinote mantiqueira*	Pico do Gavião, Andradas ‐ MG	mPG	Poços de Caldas Plateau	GBS	
ALA PG‐3	*Actinote mantiqueira*	Pico do Gavião, Andradas ‐ MG	mPG	Poços de Caldas Plateau	GBS	
ALA PG‐4	*Actinote mantiqueira*	Pico do Gavião, Andradas ‐ MG	mPG	Poços de Caldas Plateau	GBS	
ALA PG‐5	*Actinote mantiqueira*	Pico do Gavião, Andradas ‐ MG	mPG	Poços de Caldas Plateau	GBS	
ALA PG‐6	*Actinote mantiqueira*	Pico do Gavião, Andradas ‐ MG	mPG	Poços de Caldas Plateau	GBS	
ALA PG‐7	*Actinote mantiqueira*	Pico do Gavião, Andradas ‐ MG	mPG	Poços de Caldas Plateau	GBS/ Barcode	PP885773
ALA PG‐8	*Actinote mantiqueira*	Pico do Gavião, Andradas ‐ MG	mPG	Poços de Caldas Plateau	GBS	
ALA PG‐9	*Actinote mantiqueira*	Pico do Gavião, Andradas ‐ MG	mPG	Poços de Caldas Plateau	GBS/ Barcode	PP885774

Total genomic DNA (gDNA) was purified using the CTAB protocol (Doyle & Doyle, 1987), modified after Silva‐Brandão et al. (2018). Total gDNA was eluted with 50 μL of AE buffer, quantified using a Qubit 4 Fluorometer (ThermoFisher Scientific, Waltham, MA, USA), and stored at −20°C. Gel electrophoreses were performed with 1% agarose gel in 50 mM Tris‐acetate (TAE buffer), using 5 μL of each sample to check extraction quality. The ratios of the wavelengths 280/260 and 260/230 were estimated with a NanoDrop UV spectrophotometer (ThermoFisher Scientific).

### 2.2 Construction of libraries and sequencing

Genotyping‐by‐sequencing libraries were constructed using standard protocols (Poland et al., 2012), with minor modifications, at the Plateforme d'Analyses Génomiques of the Institut de Biologie Intégrative et des Systèmes (IBIS, Université Laval, Québec City, Canada). Total gDNA (200 ng) was digested with both high‐fidelity enzymes PstI and MspI (New England Biolabs, Ipswich, MA, USA). Three libraries were prepared in total, each one with 96 pooled individuals. One of 96 barcoded adapters was attached to the PstI cut site for each sample and a common adapter (adapter 2) was attached to the MspI cut site of all samples using T4 ligase (New England Biolabs). Each one of the 96 samples (same volume) was pooled and the mixture was size‐selected using a 2% agarose gel cassette on a BluePippin instrument (Sage Science, Beverly, MA, USA), with the elution time set from 50 to 65 min. Eluted fragments were used for multiplexed polymerase chain reactions (PCR), using standard forward primer A and reverse primer C. Final libraries were checked for quality on a High Sensitivity BioAnalyzer chip (Agilent, Santa Clara, CA, USA) and quantified using Picogreen (Promega, Sunnyvale, CA, USA). Each library was sequenced in one lane of an Illumina HiSeq 4000 SR100 (San Diego, CA, USA) using 100‐bp single‐end reads at the McGill University and Génome Québec Innovation Centre (Montreal, Canada).

### 2.3 Quality checking and demultiplexing

The quality of the reads was checked using FastQC. To summarize all the FastQC reports, the tool MultiQC (Ewels et al., 2016) created a single report to visualize the output. After quality checking, demultiplexing and SNP calling were performed using a script with specific parameters on the software Stacks v. 2.62 (Catchen et al., 2013). Demultiplexing raw reads with process_radtags tool allowed to: demultiplex; remove adapter sequences and the remainder of the restriction sites; correct or remove sequences with ambiguous barcodes and filter out reads with a raw Phred score of less than 10.

### 2.4 Optimization of stacks parameters

The protocol established by Paris et al. (2017) to identify the optimal parameters for the “de novo” analysis was followed. This optimization is critical due to the comparison of two different species, which have higher variability than intraspecific samples. A sub‐sample of one individual from each population was sorted at random to obtain the greatest genetic diversity from the total sampling. For this shortened data set, the Stacks protocol was performed several times, varying only one parameter in each run. Initially, the standard parameters: *M*—the maximum distance (in nucleotides) allowed between stacks (default 2); *m*—the minimum depth of coverage required to create a stack (default 3); *N*—maximum distance allowed to align secondary reads to primary stacks (default: *M* + 2). Then, the parameters of the ustacks program were increased: *m* from 3 to 4 (*m*_3_–*m*_4_) and the *M* parameter from 2 to 5 (*M*_2_–*M*_5_). In addition to these parameters, the cstacks parameter *n* from 1 to 5 (*n*1–*n*5) was tested while all other parameters (*m*_3_, *M*_2_, and *n*_1_) remained constant. The parameters were chosen to maximize the number of recovered polymorphic loci and were selected when further increases in the parameter resulted in the same number of recovered polymorphic loci. To incorporate the interspecific polymorphisms, the populations parameter was set to −*R* = 90%, indicating that SNPs were present in 90% of the samples, including at least one *A. alalia* individual.

### 2.5 SNPs calling and population statistics

Firstly, all loci were recovered in each sample using ustacks (optimized parameters: ‐M 4, ‐m 3), which aligned the short‐read sequences into exactly matching stacks (or putative alleles). A catalog of loci was created in the cstacks (parameter: −*n* 5), merging the alleles of all samples to form a consensus without a reference genome. Following, samples were matched back against the catalog using the option sstacks and transposing the data (so it is oriented by locus instead of by sample) with tsv2bam. For de novo analyses and single‐end reads, the final step was to call SNPs from the loci with the gstacks program.

The *population* program (parameters—write_single_snp‐R 50 ‐p 6) was used to compute standard population genetics statistics (expected/observed heterozygosity, *π*, and *F*_IS_), as well as to export a variety of standard output formats (Catchen et al., 2013). We calculated divergence from Hardy‐Weinberg equilibrium (HWE) for each locus and estimated SNP and haplotype‐based *F* statistics.

### 2.6 Phylogenetic reconstruction

The W‐IQ‐TREE tool (Trifinopoulos et al., 2016) was used to reconstruct a maximum likelihood (ML) phylogenetic hypothesis, with two objectives: to ascertain whether there is a phylogenetic structure between the species and whether populations (localities) have a phylogenetic structure. The matrix was composed of 142 individuals and 9370 SNPs, 6550 of which were parsimony‐informative markers, and 2821 singleton sites. Four individuals were removed from the original matrix due to more than 50% missing values. Two other species from the *Actinote*'s orangish‐red mimicry complex were used as outgroup: *A. catarina*, the putative sister species of the *A. alalia* + *A. mantiqueira* clade, and *A. dalmeidai*. The program ModelFinder (Kalyaanamoorthy et al., 2017) was applied to determine the best substitution model according to the Bayesian information criterion (BIC), including an ascertainment bias correction (+ASC) model (Lewis, 2001), as suggested for alignments without constant sites (such as SNP data). In this way, the ML analysis was run under the model TVMe+ASC+G4. Support for nodes was evaluated with 5000 Shimodaira‐Hasegawa‐like (SH‐aLRT) and ultrafast bootstrap (UFBoot2) approximations (Guindon et al., 2010; Hoang et al., 2017).

### 2.7 Population genetic structure

Genetic clustering and admixture analyses of populations of *A. alalia* and *A. mantiqueira* were performed using three alternative methods to identify population structure. The matrix of SNPs generated in the *population* program was considered for these methods, with the sampling locations as groups. The Discriminant Analysis of Principal Components (DAPC) was performed to estimate the genetic structure in the R package adegenet (Jombart, 2008). The number of retained PCs was estimated by the a‐score to optimize the trade‐off between the power of discrimination and overfitting of the data. Identification of the clusters was achieved by the find.clusters function, and the number of clusters (*K*) was evaluated regarding the Bayesian Information Criteria. To detect structuring among the populations of *A. mantiqueira*, *K* values close to the optimum were also investigated (Appendix S1, Table S1).

The second method used to infer population structure used sparse, a non‐negative matrix factorization (sNMF) to estimate individual fly admixture coefficients implemented in the R package LEA with the *snmf* function (Frichot & François, 2015). The estimates of ancestry coefficients are similar to the program STRUCTURE (Pritchard et al., 2000) for out‐crossing species, and the estimates can be more accurate in the presence of inbreeding (Frichot et al., 2014). The sNMF algorithm tested *K* values from 1 to 8, with 200 repetitions per *K* value and other options set to default values in all cases. The best fit value of *K* was selected using the cross‐entropy criterion as detailed in the manual of the LEA package.

The third method incorporated spatial information to inform individual ancestry estimates using the R package Tess3r (Caye et al., 2016). We used the default values of the program, except for the maximum number of iterations of the optimization algorithm, which was increased to 1000. The optimal value of *K* is inferred when the cross‐validation curve exhibits a plateau or starts increasing, but the cross‐validation criterion did not exhibit a minimum value or a plateau (Appendix S1, Table S1). Therefore, the *K* values obtained in the other cluster analyses (DAPC and sNMF) were investigated.

Overall genetic structure was estimated by a nonhierarchical analysis of molecular variance (AMOVA) using the software Arlequin v. 3.5 (Excoffier & Lischer, 2010). Hierarchical AMOVA was conducted among: (i) *A. alalia* and *A. mantiqueira* and (ii) clusters of sampling localities identified by the three clustering methods (DAPC, sNMF, Tess3r). Genetic structure was interpreted from the *Φ* statistics associated with different hierarchical levels at which variation is distributed (Excoffier et al., 1992). The significance of the Φ_ST_ values was evaluated using 10,000 permutations, a computed distance matrix using pairwise difference, and gamma value = 0. Slatkin pairwise *F*_ST_ values were also estimated in Arlequin (Slatkin, 1995).

### 2.8 Obtaining sequences from the COI barcode region

The barcode region (Hebert et al., 2003), which is the 5′ end of the mitochondrial DNA (mtDNA) gene cytochrome oxidase subunit I (COI, ca. 658 bp), is widely used in the delimitation of butterfly species (Silva‐Brandão et al., 2009). Hence, to assess the species limit between *A. alalia* and *A. mantiqueira* with this traditional molecular marker, 59 barcode sequences were investigated, including all samples available on GenBank (Benson et al., 2005) and BOLD (Ratnasingham & Hebert, 2007). The barcode region was amplified from 39 samples, which were also evaluated with GBS libraries (Table 1). In total, these specimens represent 13 sampling localities (collection data and GenBank accession codes are shown in Table 1). The barcode region was amplified using the primers LCO (F) and NANCY (R) (Caterino & Sperling, 1999; Folmer et al., 1994), following the procedures described in Magaldi et al. (2021). Purified PCR products were sequenced using both forward and reverse primers in a 3730xl DNA Analyzer (ThermoFisher Scientific).

### 2.9 COI gene analyses

To detect the COI haplotypic structure of *A. alalia* and *A. mantiqueira*, a haplotype network was designed using PopART with the TCS network option (Leigh & Bryant, 2015). To propose a phylogenetic hypothesis, a Bayesian inference (BI) was carried out using MrBayes v. 3.2 (Ronquist et al., 2012) on the CIPRES portal (Miller et al., 2010), including one individual of *A. catarina* as an outgroup (Table 1). The model‐jumping feature was employed to select the best nucleotide substitution model (Ronquist & Huelsenbeck, 2003). The model with the highest posterior probability was the GTR submodel [121131]. The gamma parameter was also included to allow site rate variation. Four simultaneous chains were run for 10^7^ generations in two runs, sampling trees every 1000 cycles. The first 25% of trees were discarded as “burn‐in.” The convergence of the likelihood traces of the independent runs was assessed with TRACER v1.5 (Rambaut et al., 2018), and the ESS (effective sample size) values were verified to be above 3700 for all parameters, which indicates that they were sufficiently sampled to estimate their posterior distributions (Drummond et al., 2006).

### 2.10 Species delimitation analyses

The genetic distance between individuals was calculated using two matrices: one containing the COI barcode sequences and the other with the SNPs obtained in the GBS analysis. They were estimated in Mega X (Kumar et al., 2018) using the substitution model Kimura‐2‐Parameters (K2P; Kimura, 1980) with the pairwise deletion option, which removes sites containing missing data or alignment gaps from the analysis as the need arises. We compared the results obtained with the different molecular markers to evaluate the efficiency of SNPs markers with the commonly used markers in molecular taxonomy studies (COI barcode region). Three methods for molecular species delimitation were employed to test the sensitivity between them, checking if the proposed taxonomic delimitation would be found. The Automatic Barcode Gap Discovery (ABGD) (Puillandre et al., 2012), based on the COI alignment, was applied using the mean genetic distances calculated under the K2P substitution model, with default options, through the online graphic version (https://bioinfo.mnhn.fr/abi/public/abgd/abgdweb.html). The PTP and bPTP methods (Zhang et al., 2013) were carried out using the tree reconstructed with the COI sequences and executed for 10^5^ generations with a thinning of 100 and “burn‐in” of 0.1. These analyses were performed using the online server of Exelixis Labs (http://species.h‐its.org/).

### 2.11 Paleoclimatic distribution simulations

Ecological niche modeling (ENM) was used to test the influence of paleoclimate on the distribution of *A. mantiqueira* and *A. alalia*. The occurrence points from each species were obtained from an extensive dataset (Gueratto, 2023) and mapped in a grid cell with 5 arc‐minutes (~10 km). As result, a total of 21 occurrence points were recorded for *A. alalia*, and 78 for *A. mantiqueira*. The number of occurrences of the species at each gridded cell was standardized to avoid oversampling of specific environmental conditions (abundance biases).

In order to understand the pattern of spatial expansion and retraction of both *Actinote* species over glacial cycles, the models were created from the present to 800 thousand years ago (kya), a period that covered 19 glacial‐interglacial cycles. The climate variables of the Last Glacial Maximum (21 kya), Last Interglacial Maximum (LIG – 130 kya) and Upper Pleistocene (~787 kya) periods were obtained from the Paleoclim database (http://www.paleoclim.org/) (Brown et al., 2018; Fordham et al., 2017). To avoid collinearity between the climate variables, five axes were selected through principal component analysis (PCA), accounting for the greatest variation in temperature and precipitation from the current climate data in the Neotropical region. The coefficients of the PCA were then applied to find the axes' scores from the past climates with the purpose of maintaining the dimensionality among the climate predictors (see the protocols in do Amaral et al., 2021; Legendre & Legendre, 1998; Manly, 1994). The climate conditions (PCA axes) were interpolated by using the global stacked δ18 Oxygen curve (Lisiecki & Raymo, 2005) as a covariate (see do Amaral et al., 2021; Lawing & Polly, 2011). Interpolation was calculated each 1000 years between present time and 600 kya; and each 2000 years in the interval from 602 to 786 kya.

As each algorithm present distinct prediction according to the different niche breadth of the species (Qiao et al., 2015), their combined use may increase the accuracy of predictions by considering different niche tolerances in the potential distribution of species (Araújo & New, 2007; Diniz‐Filho et al., 2009). Therefore, five different mathematical algorithms were used: Bioclim (Nix, 1986), distance method Domain (Gower distance; Carpenter et al., 1993), support vector machines (SVM; Tax & Duin, 2004), maximum entropy – Maxent (Phillips & Dudik, 2008) and Ecological‐Niche Factor Analysis (ENFA; Hirzel et al., 2002). As a way of evaluating the generated models, the occurrence points were randomized in two groups, training and testing, which contain 70% and 30% of the occurrence points, respectively. The model's performance was evaluated with the D statistic, which considers only presence records and weights the true positive rate (TPR) by the inverse of the proportional predicted distribution area (π): *D* = TPR × (1 − π) (Pearson et al. 2007). Threshold values using maximum sensitivity and specificity were calculated in order to maximize the correctness of presences and absences. After defining the thresholds, a prediction map of each species was obtained following the ensemble technique (Araújo & New, 2007).

All modeling methods described above were followed separately to build a climate‐based to each geological period, thus resulting in 695 final consensus maps for each of the two *Actinote* species: models at each 1 ky BP (between present and 600 ky BP) and 2 ky BP (between 602 and 786 ky BP) intervals. The glacial and Interglacial periods were delimited according to Marine Isotope Stages (MIS) proposed by Lisiecki and Raymo (2005). All analyzes were performed in R 4.0.2 (R Development Core Team, 2018).

## 3 RESULTS

### 3.1 Genotyping‐by‐sequencing statistics

The number of reads for each run per lane ranged from 348 to 352 million. Sequencing quality across all bases was above 32 Phred Score, no adapters were found in the sequences, and the missing (*N*) content in all bases was not significant. After the demultiplexing, 96.7% of reads were retained, and 266,933 loci were genotyped with a mean coverage of 89.9× and a standard deviation of 35.8×.

### 3.2 Optimization of stacks parameters

Using the standard Stacks parameters, 9971 polymorphic loci were obtained, with 8126 SNPs present in 90% of our sub‐sample of 12 individuals. Increasing the ‐m parameter, which is the minimum coverage required to produce a new stack, led to a slight reduction in polymorphic loci (R 90% = 9604 loci). Therefore, for all the following analyses (including the optimized one), the value of −*m* = 3 was applied, which is the default of the ustacks program. Similarly, increasing the −*M* parameter, which is the maximum permitted variance in the same stack, from its initial standard value (−*M* = 2) to −*M* = 3, resulted in a non‐significant rise in the number of polymorphic loci (R 90% = 9894 loci). However, the number of observed SNPs increased to 8251 with a value of −*M* = 4. With the increment to −*M* = 5, there was no increase in the number of polymorphic loci, or even in the number of SNPs, so we maintained the value of −*M* = 4 in the following analyses.

As expected for data obtained from two different species, the parameter that most increased the number of polymorphic loci was −*n*, which determines the number of “errors” allowed between samples when the catalog is built. In highly variable populations or between different species, there is a greater chance that the same locus will be divided into more than one stack (or alignment) in “de novo” analyses due to interspecific or interindividual variation. The default value of −*n* is 1, which means that if more than one “error” or mutation is present in the same locus (100 base pairs), it is considered a different locus. If this is the case, the locus is divided into two stacks, decreasing the number of SNPs and increasing the number of nonvariable loci. With the increase of −*n* to 5, and the maximum for the −*M* = 4 recommended by Paris et al. (2017), 12,460 polymorphic loci were recovered, for a total of 11,178 SNPs. Therefore, this value of −*n* was used for the analysis of all samples.

### 3.3 Population genetic statistics

After loci filtering using the *population* program, 18,182 loci were kept, composed of 1,696,358 sites with 16,796 variant sites (SNPs). The final matrix used in all downstream analyses was then composed of 135 individuals of *A. mantiqueira* and 6 individuals of *A. alalia*. Most sequenced SNPs (10,884, 64.8%) were in Hardy–Weinberg equilibrium (*p* < .05), while three of the populations have more than 1000 SNPs out of HWE. The population with the highest mean nucleotide diversity and inbreeding coefficient (*F*_IS_) is Delfim Moreira (“mDM”).

The Slatkin's pairwise *F*_ST_ values ranged from 0 to 0.45 (“aBJ”, *A. alalia* × “mMV”, *A. mantiqueira*) (Table 2). The mean *F*_ST_ within populations of *A. mantiqueira* was 0.0122 ± 0.0136. Interspecific *F*_ST_ varied from 0.30 (“aFL” × “mBoc”) to 0.45 (“aBJ” × “mMV”), with a much higher average (0.3685 ± 0.035) than the *F*_ST_ between *A. mantiqueira* populations.

**TABLE 2:** Slatkin's pairwise *F*_ST_ values calculated from the SNPs for *Actinote alalia* (*a* localities) and *A. mantiqueira* (*m* localities).

	mSB	mDM	mPG	mFX	mI	mCJ	mMV	mPD	mBoc	mPC	aFl
mDM	0.0015										
mPG	**0.0189**	**0.0181**									
mFX	0	0	0								
mI	**0.0168**	**0.0159**	**0.0287**	0							
mCJ	0	0.0010	**0.0176**	0	**0.0149**						
mMV	0	0	0	0	0	0					
mPD	0.0017	0.0051	0.0183	0	0.0151	0.0042	0				
mBoc	**0.0252**	**0.0257**	**0.0422**	0	**0.0400**	**0.0242**	0	0.0254			
mPC	**0.0209**	**0.0203**	**0.0277**	0	**0.0336**	**0.0193**	0	0.0187	**0.0482**		
aFl	**0.3224**	**0.3594**	**0.3635**	0.4173	**0.3713**	**0.3532**	0.4182	**0.3627**	**0.3051**	**0.3465**	
aBJ	**0.3482**	**0.3639**	**0.3816**	**0.3997**	**0.3827**	**0.3523**	**0.4506**	**0.3854**	**0.3193**	**0.3674**	0.0399

*Note*: The codes correspond to the GBS‐sampling locations in Table 1. Bold numbers are statistically significant values under *α* < 0.05.

### 3.4 Phylogenetic reconstruction hypothesis

**FIGURE 2:**
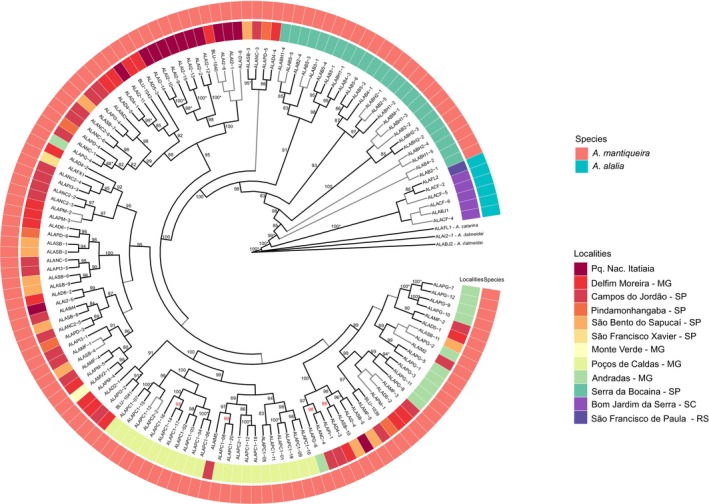
Maximum‐likelihood inference for individuals of *Actinote mantiqueira* and *A. alalia*. Branches with SH‐aLRT values >85 are presented in black. Branches with Ufboot values ≥95% have an asterisk. The layers present the species identification and the sampling locations of each individual.

The clade composed by *A. catarina*, *A. alalia* and *A. mantiqueira* exhibited strong support in the ML phylogenetic hypothesis (SH‐aLRT = 100, UFBoot2 = 100) (Figure 2). The clade composed of *A. alalia* + *A. mantiqueira* was also recovered with high support (SH‐aLRT = 100, UFBoot2 = 100) and as sister group of *A. catarina*. Both *A. alalia* (SH‐aLRT = 100, UFBoot2 = 100) and *A. mantiqueira* (SH‐aLRT = 99, UFBoot2 = 99) were recovered as reciprocally monophyletic. Intraspecific relationships within *A. mantiqueira* failed to recover the individuals from Serra da Bocaina (“mBoc”) as a clade, and they instead appear as small clades external to all remaining populations. None of the localities within the Serra da Mantiqueira were monophyletic. All individuals in the population of Poços de Caldas comprised a clade (“mPC”, SH‐aLRT = 99), although a single individual from Campos do Jordão (“mCJ”, ALAIM‐5) appears as part of this clade.

### 3.5 Population structure

**FIGURE 3:**
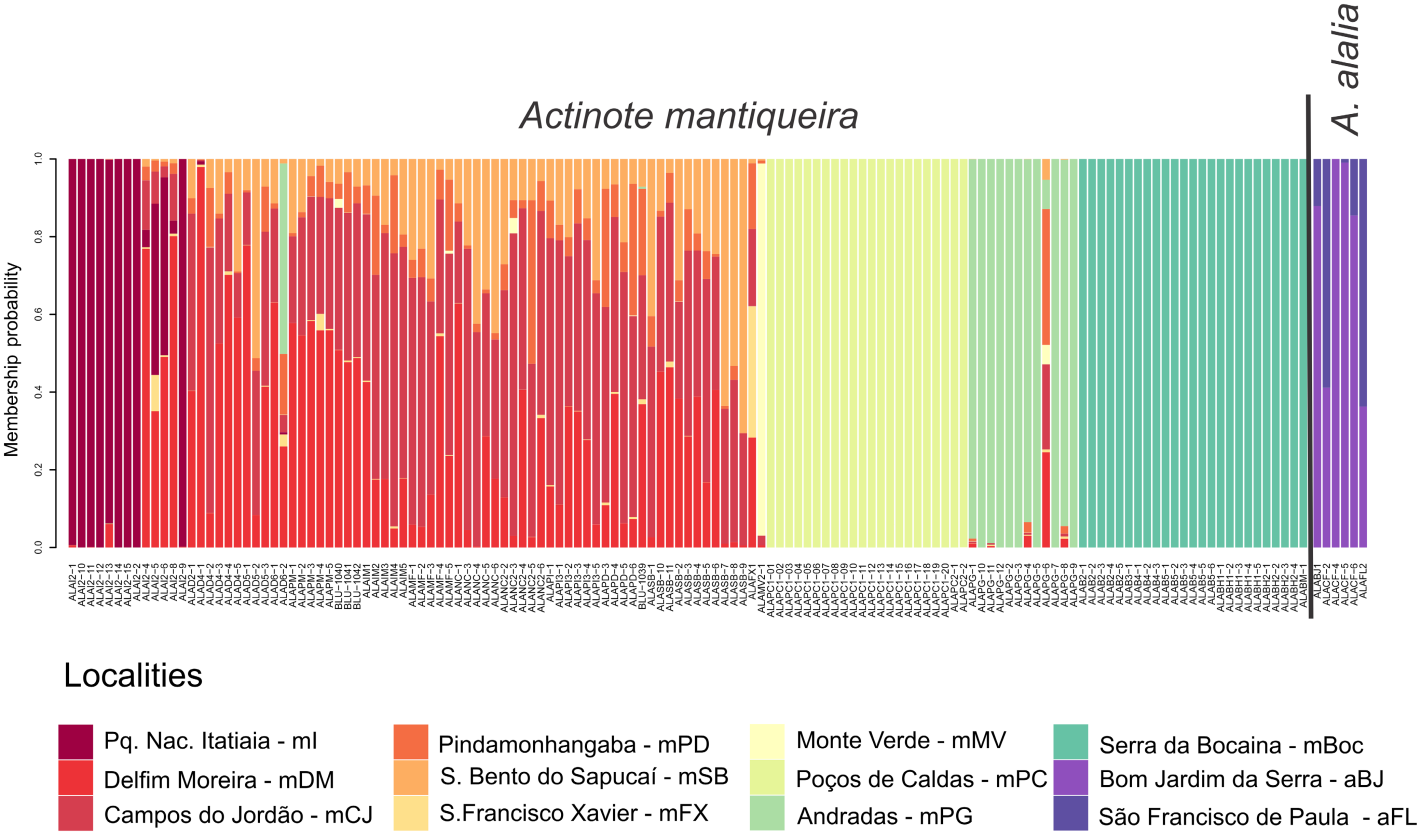
Barplot of DAPC by localities. Colors correspond to each location sampled, and the *Y*‐axis is the probability of the individual membership.

The two populations of *A. alalia* comprise one genetic cluster isolated from all populations of *A. mantiqueira* in the DAPC by location (Figure 3). All individuals from Serra da Bocaina (“mBoc”) have a high probability of belonging to one unique cluster. Similarly, all individuals collected in Poços de Caldas (“mPC”) constituted a second cluster. In addition, two populations have some structure, “mI” and “mPG”, although some individuals from these locations have mixed probabilities between four or more locations. Besides these locations, all individuals from Serra da Mantiqueira have mixed probabilities between locations, showing no geographic structure.

**FIGURE 4:**
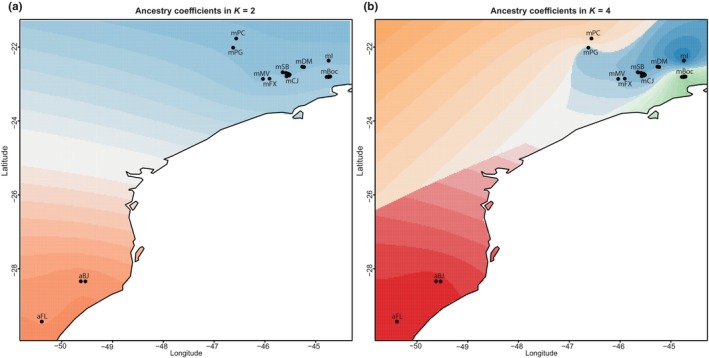
Spatial distribution of the ancestry coefficients obtained in the Tess3r package. (a) Ancestry coefficients of the two clusters (*K* = 2). Blue = *A. mantiqueira* locations; Orange = *A. alalia* locations. (b) Ancestry coefficients using four clusters (*K* = 4). Points correspond to samples' coordinates.

The best number of clusters indicated by the DAPC analyses based on BIC values was *K* = 2 (BIC = 978.6421), which perfectly defines one cluster for each species. In the sNMf analyses, the lowest cross‐entropy is also *K* = 2 and the two ancestral populations correspond to the two species as well. Additionally, the cross‐validation criterion did not show a minimum value or a plateau in the Tess3r analysis (Appendix S1, Table S1). For this reason, the *K* = 2 value was investigated, and the same clustering result as the other analyses was obtained (Figure 4).

Exploring other *K* values obtained in the DAPC analyses associated with close values of BIC, as *K* = 3 (BIC = 980.9029), an additional cluster was obtained within *A. mantiqueira*, consisting of individuals from “mBoc” (which is the only population sampled from the Serra do Mar Mountain range). Using *K* = 4 (BIC = 983.8178), the clusters detected with values of *K* = 2 and *K* = 3 were also observed, and in addition to them, a fourth group subdivides the individuals of *A. mantiqueira*. This group contains all individuals sampled at location “mPC”, which is one of the two populations obtained from the Poços de Caldas plateau. For *K* = 5 (BIC = 987.0982), the full correlation between the sampled location and cluster membership is no longer observed. The four groups obtained with the lower *K* values (2, 3, and 4) were recovered, but the fifth group is composed of four individuals from the “mI” location (of the 13 individuals sampled there) (Appendix S1, Table S1). To investigate the population structure of *A. mantiqueira* populations, the value of *K* = 4 was applied in the sNMF and Tess3r analyses. The four clusters obtained in the DAPC analyses were equally recovered (Figure 4).

Overall genetic structure inferred by a nonhierarchical AMOVA resulted in a significant *Φ*_ST_ value of 0.065 (*p* < .001). The hierarchical AMOVA considering the two species showed a *Φ*_ST_ value of 0.3243 (*p* < .0001), with 30.46% of the variation between species, 1.96% of the variation among populations within species, and 67.57% within populations. The hierarchical AMOVA considering the three clusters in *A. mantiqueira* (corresponding to Serra da Mantiqueira, Serra do Mar, and Poços de Caldas plateau) showed a *Φ*_ST_ value of 0.036 (*p* < 0.001), with 1.57% of the variation among mountain ranges, 2.04% of the variation among populations within groups, and 96.39% within populations.

### 3.6 Barcode analyses

**FIGURE 5:**
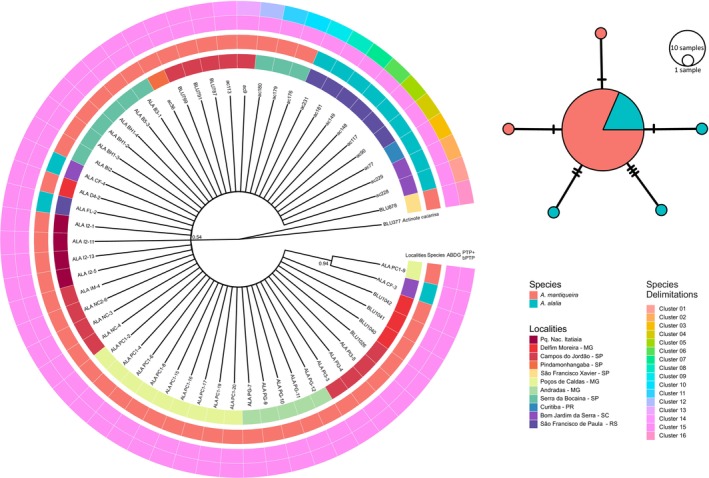
BI topology based on COI sequences from *Actinote mantiqueira* and *A. alalia*. Posterior probabilities are given on the branches (0–1). The circular layers present (from the most internal to the external): the sample localities; the species identification; the species delimitation results of ABDG, PTP, and bPTP methods (together). On the top right, the haplotype network obtained with COI sequences.

The TCS haplotype network (Figure 5) resulted in six haplotypes: the most common haplotype is shared between most individuals of the two species, three of them are exclusive for *A. mantiqueira*, and two of them are exclusive for *A. alalia*. The highest number of mutational steps found between two haplotypes was six. The BI (Figure 5) based on COI sequences failed to recover clades for the two species or sampling localities.

### 3.7 Species delimitation

**FIGURE 6:**
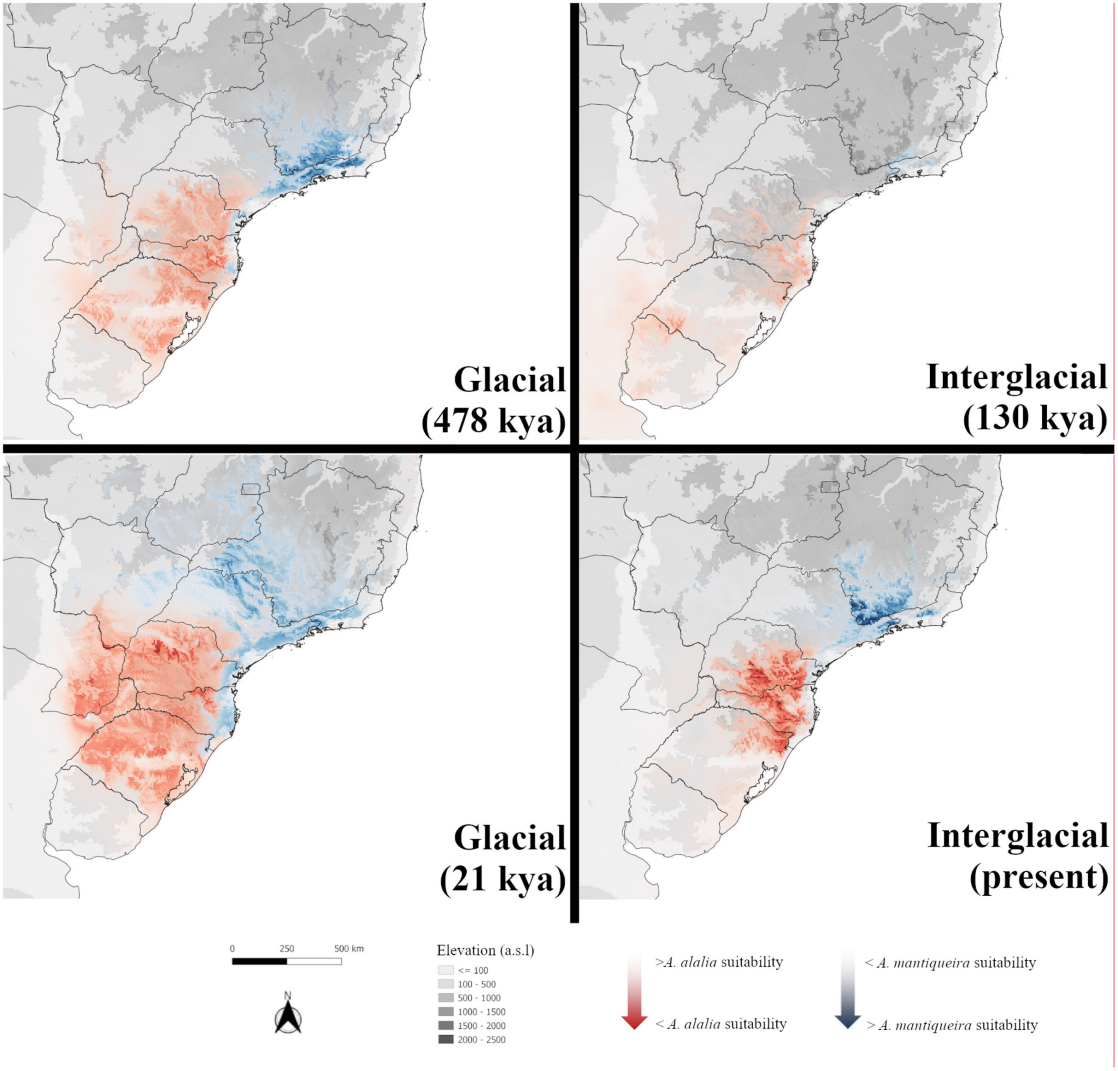
Maps of *Actinote mantiqueira* and *A. alalia* potential occurrence in glacial and interglacial periods (considering the 19 MIS), according to the Ecological Niche Modellng.

The genetic distances among all COI sequences varied from 0 to 2% (Appendix S1, Table S1). The mean intraspecific genetic distance among *A. alalia* was 0.04%, and for *A. mantiqueira*, it was 0.29%. No “barcode gap” was found between inter‐ and intraspecific genetic distances since the estimated mean interspecific distance was 0.17%. Comparatively, the genetic distances among all SNPs sequences varied from 3.16% to 35%. Regarding the genetic distances estimated with SNPs, there is a substantial difference between intraspecific (*A. alalia* = 10.72%; *A. mantiqueira* = 11.39%) and interspecific mean distances (31.52%). The ABDG method applied to the *barcode* sequences recovered only one group that comprises all individuals sampled for the two species. However, the PTP and bPTP methods (Figure 6) recovered 16 groups, a large one including individuals of both species and 15 groups with one individual each.

### 3.8 Paleoclimatic distribution simulations

The model from the present climatic scenario confirms the disjunct distribution of the species, with *A. alalia* occurring in southern Brazil (in the states of Rio Grande do Sul, Santa Catarina and Paraná), and *A. mantiqueira* occurring in southeastern mountains (in the states of São Paulo, Minas Gerais and Rio de Janeiro) (Figure 6). Paleoclimate spatial simulations show a wider spatial distribution during all 19 glacial periods for both species, with *A. alalia* expanding its potential distribution to inland areas, reaching northern Argentina and southern Paraguay, while *A. mantiqueira* had an expanded potential range in the Serra do Mar Mountain range, reaching the coastal mountains of the states of Paraná and Santa Catarina, as well as the interior of the states of São Paulo and Minas Gerais. On the other hand, both species reduce their distribution area in the mountainous regions of the Atlantic Forest in 19 interglacial periods (Figure 6). These findings show that during the glacial eras, the two species could persist in lower elevations in the region that is currently a distribution gap between them both, revealing the possible connection between both areas via the Serra do Mar Mountain range.

## 4 DISCUSSION

### 4.1 Taxonomic delimitation of species using different molecular markers

Research on the mechanisms of montane speciation in neotropical insects is scarce, and we are just now starting to comprehend how they contribute to diversity in neotropical forests. Cold‐adapted species on mountain peaks can quickly become reproductively isolated during warmer climates due to geographic isolation and niche specialization, though most of their genomes can still be shared due to recent gene flow. Indeed, while *A. mantiqueira* and *A. alalia* can be distinguished by a series of distinct morphological characteristics of adults, immature stages, and geographic distribution (Freitas et al., 2018), the COI barcode lacks sufficient interspecific differences to distinguish them. Therefore, based on barcodes, the genetic distance between individuals of these species is virtually zero, that is, there is no “barcode gap” (sensu Hebert et al., 2004) between *A. alalia* and *A. mantiqueira*. Furthermore, there is no genetic structure across locations based on COI, and all species delimitation methods evaluated failed to tell the two species apart. Some hypotheses have already been raised to explain this lack of information at the COI level, such as a lower diversification rate also found for other Acraeini species, intrinsically different rates of molecular evolution, or selective sweeps by endosymbionts, as already demonstrated for other Acraeini (Jiggins, 2003; Silva‐Brandão et al., 2021), and in fact all mechanisms can contribute to explaining the present pattern for this mitochondrial marker.

In contrast, all analyses performed using genome wide SNPs (genetic distance, phylogenetic hypothesis, clustering analyses, and *F*_ST_ statistics) recovered a clear pattern of two clades, separating individuals of *A. alalia* from all populations of *A. mantiqueira*, validating the taxonomic decision of Freitas et al. (2018). These markers have not been widely used yet in taxonomic studies (but see Bog et al., 2020; Ramírez‐Reyes et al., 2020). However, this genomic approach, including markers with a possible faster evolutionary rate, was able to reveal a more detailed and recent history of these populations, confirming their separated evolutionary histories, even though they still share mitochondrial haplotypes. The results are unaffected by the small number of *A. alalia* specimens. Indeed, despite the increased sampling of *A. mantiqueira*, no specimen of this species shared the same genetic cluster with individuals of *A. alalia*. Nevertheless, any examination of interpopulation variation within *A. alalia* is precluded by the small sample size.

### 4.2 Montane speciation

The information gathered with the present genomic data and current distribution recovered a phylogeographic pattern that can be associated with the São Paulo subtropical gap (sensu do Amaral et al., 2021), as a phylogeographic break for these montane butterflies. The role of the Atlantic Forest Mountain ranges in shaping the genetic structure of endemic species has already been observed for birds (do Amaral et al., 2021; Thom et al., 2020). However, this is the first time that this pattern has been reported for an invertebrate. A previous study with the cold‐associated bumblebees *Bombus morio* and *B. pauloensis*, which are mainly found in high‐altitude areas of MAF, failed to recover a strong structured phylogeographic pattern (Françoso et al., 2016). However, the results of the demographic analyses and paleodistribution models are consistent with a scenario of expansion during the LGM.

The speciation between *A. alalia* and *A. mantiqueira* probably occurred recently during the Quaternary, considering that the divergence between these species and its sister species, *A. catarina*, was estimated around 3 million years ago, at the end of the Pliocene (Magaldi, 2021). Pleistocene climatic oscillations have been hypothesized to have shaped the genetic structure of populations of other species currently found on the MAF (do Amaral et al., 2021; Françoso et al., 2016; Thom et al., 2020). Our results are consistent with paleoclimate, molecular, and pollen data obtained for other species, suggesting that the MAF taxa persisted or expanded during the LGM (Amaro et al., 2012; Carnaval et al., 2009; Leite et al., 2016). Accordingly, the ancestral populations of *A. alalia* and *A. mantiqueira* may have been limited to mountain tops several times in the past, yet they were likely able to explore currently unsuitable regions (mostly lowland) and hence expand their distribution during colder periods, which could have enabled a dispersal process to new mountain ranges (Paz et al., 2019). Mountain ranges provide diverse climatic conditions that allow montane species to adapt and survive the fluctuations in climate. This is made possible by the extensive variance in topography and altitudinal gradients (Brown Jr & Ab'Saber, 1979). Furthermore, the low levels of genetic differentiation (as estimated by the *F*_ST_ values) between the populations may not be due to high levels of current gene flow, but rather to this recent divergence (Palsbøll et al., 2004). Combining molecular and niche modeling data, we propose that Pleistocene climatic variation led to an allopatric and recent speciation process between the mountains of the Atlantic Forest.

### 4.3 The population structure of *A. mantiqueira* between mountains ranges and implications for conservation

The best number of clusters detected by the clustering analyses for all samples was *K* = 2. This *K* value, however, did not indicate any population structure for *A. mantiqueira* through its distribution. Nonetheless, phylogenetic inference and DAPC analysis revealed some intraspecific structure, implying that a population structure exists that was not detected by cluster analyses using the optimal *K* = 2. All methods proposed to determine genetic structure a posteriori will face detection limits when differentiation between groups is low, and some factors such as uneven sampling and the number of individuals may affect the optimal *K* value (Miller et al., 2020). In simulations to test the efficiency of DAPC to assess the population genetic structure, it was observed that there is a considerable inaccuracy in the face of low levels of differentiation (*F*_ST_ values < 0.1) (Miller et al., 2020). This may explain the inability to detect intraspecific population structure in *A. mantiqueira* since the mean *F*_ST_ within populations is 0.0122.

Clustering methods depict a simplified version of a complicated reality that does not always correspond to existing population genetic models (Jombart, 2012). For this reason, it is important to interpret clustering results with caution and consider additional genetic and demographic factors that may influence population structure. Additionally, incorporating information from multiple clustering methods or conducting further analyses can provide a more comprehensive understanding of genetic diversity within populations. Therefore, other *K* values were investigated, and the *K* = 4 value recovered the same clustering pattern in all analyses, regardless of the different assumptions underlying these methods. Furthermore, this clustering pattern was preferred because it is congruent with *A. mantiqueira* mountain range distribution, implying biological relevance. Indeed, a process of differentiation between the populations from Serra da Bocaina and the inland populations may be ongoing, as evidenced by the slight variations in wing patterns that exist between the two.

The phylogeographic break recovered between Serra da Mantiqueira and Serra do Mar Mountain ranges has been observed in Atlantic Forest orchids, and dispersal is a more plausible explanation than vicariance for the origin of this population structure (Pinheiro et al., 2013). The lowland regions between these mountains were potentially phylogeographic barriers to dispersal for endemic birds as well (Chaves et al., 2015).

Singularly, the sample of *A. mantiqueira* from Poços de Caldas (MG) stands out from the rest of the Serra da Mantiqueira. The Poços de Caldas plateau is a circular structure of Mesozoic age comprising a suite of alkaline volcanic and plutonic rocks with elevations up to 1500–1600 m above sea level in its borders. Its original vegetation coverage consisted of the Atlantic Forest biome, but this region has suffered high degradation, losing areas of native vegetation to pastures (Grohmann et al., 2007; RadamBrasil., 1983; Schorscher & Shea, 1992). The highlands of Poços de Caldas also have a distinct geology when compared to the rest of the Serra da Mantiqueira complex (Schorscher & Shea, 1992), making them a distinct and representative area of endemism for the region's anurans (Neves et al., 2018). Because of the strong influence of a different domain (the Cerrado savannas) in this location, phytophisiognomy factors may explain some of the observed genetic discontinuity. Furthermore, this location is one of the most geographically distant from the others in Serra da Mantiqueira and may be under greater isolation by distance (IBD) effect.

The discontinuity between mountain ranges may be partially due to historical processes, such as the founder effect, which involves the colonization of new mountains through dispersal. By this model, new populations would be established by few individuals from a large ancestral population, suffering the effects of genetic drift after establishment and large changes in allele frequencies due to sampling error (Templeton, 2008). Another potential outcome is the reduction in genetic diversity caused by genetic drift following the disruption of gene flow between distinct mountain ranges. In this case, the ancestral population would have been distributed over the entire sampled extent, and later, climatic barriers during interglacial periods may have caused isolation in sub‐populations. Natural selection may also have influenced these polymorphisms in different locations, selecting those more adapted to the ecological, climatic, and niche variations of these mountain ranges, promoting faster differentiation among populations (Matsubayashi & Fujiyama, 2016). Our results suggests that gene flow between populations of *A. mantiqueira* may be limited, leading to potential genetic divergence within the species. Therefore, the same factors that lead to allopatric speciation in *A. mantiqueira* and *A. alalia* may also operate at the intrapopulation level in *A. mantiqueira*.

Climate change is a potential danger to the survival of these species that inhabit mountainous regions. This is particularly true for the Atlantic Forest, a region of high biodiversity that is currently facing habitat fragmentation as a result of urbanisation and human activities, leaving only 12% of its original vegetation intact. (SOS Mata Atlântica, 2019). According to the niche models generated in the present study, the fragments suitable for these species will become scarcer as the mean temperature rises. Habitat fragmentation may reduce dispersal and, consequently, genetic connectivity among these populations (Cunningham & Moritz, 1998; Dayanandan et al., 1999; Gerlach & Musolf, 2000). This major landscape alteration is likely to change gene flow and promote genetic drift in natural populations (Dixo et al., 2009; Vandergast et al., 2006). The loss of genetic connectivity may be detrimental to long‐term species persistence (Gilpin & Soulé, 1986; Templeton et al., 1990). For this reason, more phylogeographic, climate modeling, and demographic studies on the fauna of MAF will be crucial for the conservation of the biodiversity of this hotspot biome in scenarios of habitat fragmentation and climate change.

### 4.4 General conclusions

The two studied butterflies' species, *A. mantiqueira* and *A. alalia*, are genetically isolated entities, and the barcode molecular marker was unsuited to delimit them. The genomic data and niche modeling point to an allopatric speciation process between montane regions, in which Pleistocene climatic variation created a discontinuity of suitable habitat among the ancestral populations of the Atlantic Forest's South and Southeast regions. During glacial periods, the Serra do Mar Mountain range may have been a dispersal route between populations in the South and Southeast that are currently disconnected. There is a low gene flow between the populations of *A. mantiqueira* in the two montane regions of the Southeast, the Serra do Mar and the Serra da Mantiqueira, evidencing that the valleys between these mountain ranges are effective barriers for the dispersal of this species. According to the findings of this study, topography and climatic variation played an important role in *Actinote* butterfly diversification.

## AUTHOR CONTRIBUTIONS

**Luiza de Moraes Magaldi:** Formal analysis (lead); investigation (lead); writing – original draft (lead); writing – review and editing (equal). **Patrícia Eyng Gueratto:** Formal analysis (supporting). **Enrique Ortega‐Abboud:** Formal analysis (supporting). **Thadeu Sobral‐Souza:** Formal analysis (supporting). **Mathieu Joron:** Funding acquisition (supporting); methodology (supporting); writing – review and editing (supporting). **Anete Pereira de Souza:** Funding acquisition (supporting); resources (supporting); writing – review and editing (supporting). **André Victor Lucci Freitas:** Conceptualization (equal); funding acquisition (supporting); investigation (equal); methodology (equal); supervision (supporting); writing – review and editing (equal). **Karina Lucas Silva‐Brandão:** Conceptualization (lead); data curation (equal); funding acquisition (lead); investigation (equal); methodology (lead); project administration (lead); resources (lead); supervision (lead); writing – original draft (equal); writing – review and editing (lead).

## CONFLICT OF INTEREST STATEMENT

The authors declare that the disclosed information is correct and that no other situation of real, potential or apparent conflict of interest is known. We undertake to inform you of any change in these circumstances, including if an issue arises during the course of the meeting or work itself.

## Supporting information


Appendix S1.

Table S1.

## Data Availability

Raw sequence reads and metadata are deposited on the NCBI Nucleotide Database (BioProject ID: PRJNA1122554; BioSamples: SAMN41787525 ‐ SAMN41787670).
