# Supplementary material for: Montane diversification as a mechanism of speciation in neotropical butterflies
Source: Ecol Evol. 2024 Jul 11;14(7):e11704. doi: 10.1002/ece3.11704 (PMC11239956; doi:10.1002/ece3.11704)

Appendix 2. a) Number of clusters (K) versus BIC value in DAPC find.clusters function; b) sNMF cross-entropy criterion versus number of ancestral populations (K); c) Tess cross-validation criterion versus number of ancestral populations (K).

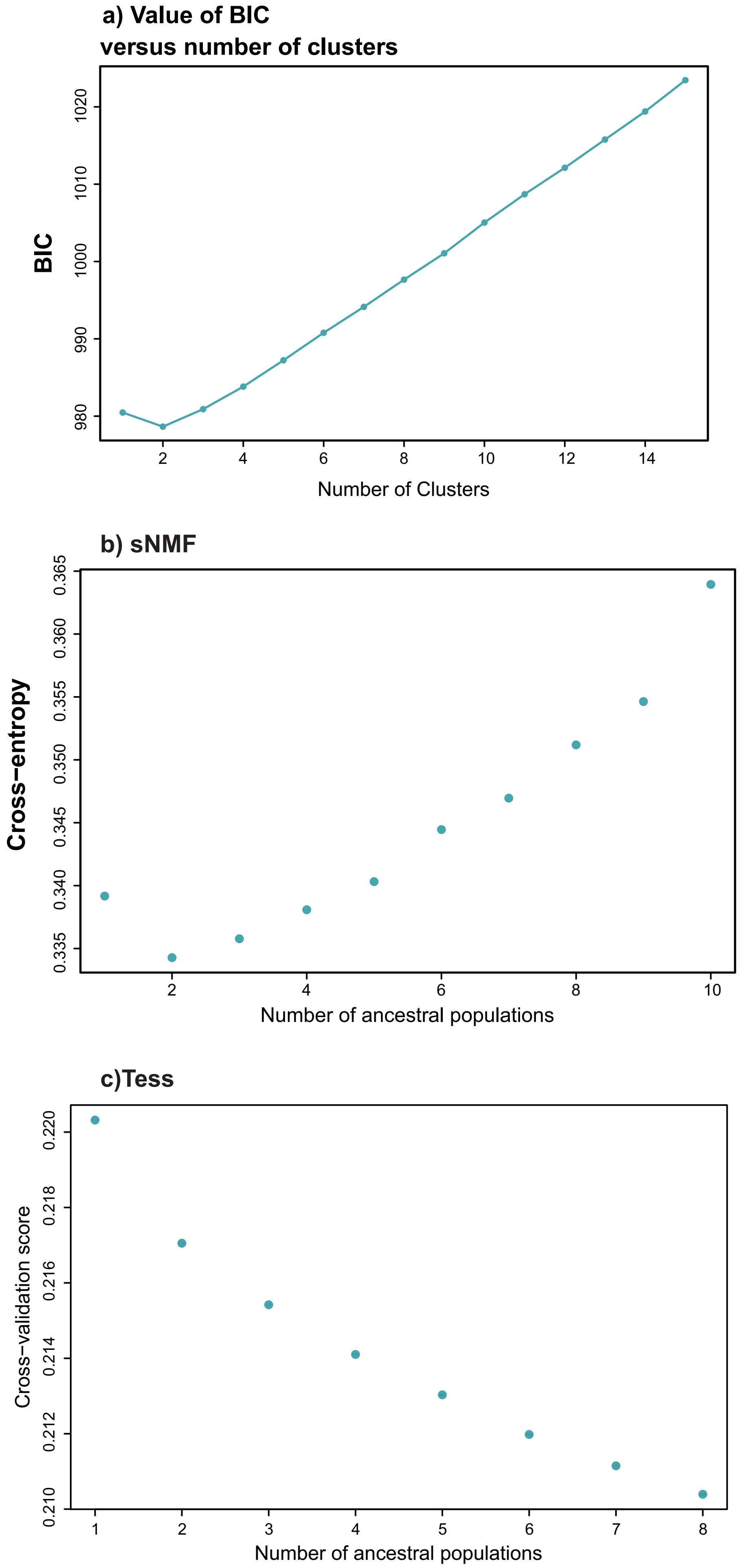

Supplement: Supplementary file 1 — Appendix S1. [file ECE3-14-e11704-s001.pdf]
