# Supplementary material for: Montane diversification as a mechanism of speciation in neotropical butterflies
Source: Ecol Evol. 2024 Jul 11;14(7):e11704. doi: 10.1002/ece3.11704 (PMC11239956; doi:10.1002/ece3.11704)
Supplement: Supplementary file 2 — Table S1. [file ECE3-14-e11704-s002.docx]

**Table S1.** Summary of the standard set of population genetic statistics across *A. alalia* (*a* localities) and *A. mantiqueira* (*m* localities) species localities. Population Code (Pop. Code) correspond to the GBS-sampling locations on Table 1; number of private alleles in this population; number of loci out of HW-equilibrium; mean number of individuals per locus in this population; variance (σ2); Standard Error (S_E_); mean frequency of the most frequent allele at each locus in this population (P).

| Pop. Code | Private alleles | Out of HW-equilibrium | Nº Indv.  per locus | σ^2^ | S_E_ | P | σ^2^ | S_E_ |
| --- | --- | --- | --- | --- | --- | --- | --- | --- |
| aBJ | 1173 | 44 | 3.85 | 1.62 | 0.01 | 0.9327 | 0.0174 | 0.0011 |
| aFL | 153 | 0 | 1.00 | 0.00 | 0.00 | 0.9554 | 0.0203 | 0.0013 |
| mBoc | 746 | 1217 | 21.24 | 11.08 | 0.03 | 0.9211 | 0.0156 | 0.0010 |
| mCJ | 877 | 1230 | 21.86 | 12.14 | 0.03 | 0.9250 | 0.0146 | 0.0009 |
| mDM | 713 | 1002 | 15.98 | 6.02 | 0.02 | 0.9244 | 0.0148 | 0.0009 |
| mFX | 34 | 0 | 1.00 | 0.00 | 0.00 | 0.9564 | 0.0199 | 0.0012 |
| mI | 425 | 599 | 11.25 | 3.26 | 0.01 | 0.9271 | 0.0153 | 0.0010 |
| mMV | 38 | 0 | 1.00 | 0.00 | 0.00 | 0.9529 | 0.0213 | 0.0012 |
| mPC | 659 | 992 | 19.39 | 8.02 | 0.02 | 0.9268 | 0.0148 | 0.0009 |
| mPD | 169 | 47 | 4.19 | 0.90 | 0.01 | 0.9303 | 0.0167 | 0.0010 |
| mPG | 503 | 432 | 10.37 | 2.91 | 0.01 | 0.9279 | 0.0149 | 0.0009 |
| mSB | 449 | 349 | 9.60 | 2.23 | 0.01 | 0.9258 | 0.0151 | 0.0010 |

| Pop. Code | Obs. Het. | σ^2^ | S_E_ | Obs. Hom. | σ^2^ | S_E_ |
| --- | --- | --- | --- | --- | --- | --- |
| aBJ | 0.0880 | 0.0372 | 0.0016 | 0.9120 | 0.0372 | 0.0016 |
| aFL | 0.0892 | 0.0812 | 0.0026 | 0.9108 | 0.0812 | 0.0026 |
| mBoc | 0.0937 | 0.0211 | 0.0011 | 0.9064 | 0.0211 | 0.0011 |
| mCJ | 0.0906 | 0.0197 | 0.0011 | 0.9094 | 0.0197 | 0.0011 |
| mDM | 0.0922 | 0.0213 | 0.0011 | 0.9078 | 0.0213 | 0.0011 |
| mFX | 0.0872 | 0.0796 | 0.0024 | 0.9128 | 0.0796 | 0.0024 |
| mI | 0.0906 | 0.0242 | 0.0012 | 0.9094 | 0.0242 | 0.0012 |
| mMV | 0.0941 | 0.0853 | 0.0025 | 0.9059 | 0.0853 | 0.0025 |
| mPC | 0.0892 | 0.0211 | 0.0011 | 0.9109 | 0.0211 | 0.0011 |
| mPD | 0.0923 | 0.0344 | 0.0014 | 0.9077 | 0.0344 | 0.0014 |
| mPG | 0.0911 | 0.0242 | 0.0012 | 0.9089 | 0.0242 | 0.0012 |
| mSB | 0.0932 | 0.0241 | 0.0012 | 0.9068 | 0.0241 | 0.0012 |

* Mean observed heterozygosity in this population (Obs. Het.); mean observed homozygosity in this population (Obs. Hom.).

| Pop. Code | | Exp. Het. | σ^2^ | | S_E_ | | Exp. Hom. | σ^2^ | | S_E_ | |
| --- | --- | --- | --- | --- | --- | --- | --- | --- | --- | --- | --- |
| aBJ | 0.0906 | | 0.0267 | 0.0013 | | 0.9094 | | 0.0267 | 0.0013 | |  |
| aFL | 0.0446 | | 0.0203 | 0.0013 | | 0.9554 | | 0.0203 | 0.0013 | |  |
| mBoc | 0.1141 | | 0.0253 | 0.0012 | | 0.8859 | | 0.0253 | 0.0012 | |  |
| mCJ | 0.1096 | | 0.0235 | 0.0012 | | 0.8905 | | 0.0235 | 0.0012 | |  |
| mDM | 0.1102 | | 0.0239 | 0.0012 | | 0.8898 | | 0.0239 | 0.0012 | |  |
| mFX | 0.0436 | | 0.0199 | 0.0012 | | 0.9564 | | 0.0199 | 0.0012 | |  |
| mI | 0.1045 | | 0.0249 | 0.0012 | | 0.8955 | | 0.0249 | 0.0012 | |  |
| mMV | 0.0471 | | 0.0213 | 0.0012 | | 0.9529 | | 0.0213 | 0.0012 | |  |
| mPC | 0.1061 | | 0.0241 | 0.0012 | | 0.8940 | | 0.0241 | 0.0012 | |  |
| mPD | 0.0965 | | 0.0266 | 0.0013 | | 0.9036 | | 0.0266 | 0.0013 | |  |
| mPG | 0.1041 | | 0.0246 | 0.0012 | | 0.8959 | | 0.0246 | 0.0012 | |  |
| mSB | 0.1071 | | 0.0247 | 0.0012 | | 0.8929 | | 0.0247 | 0.0012 | |  |

* Mean expected heterozygosity in this population (Exp. Het.); mean expected homozygosity in this population (Exp. Hom.).

| Pop. Code | π | σ^2^ | S_E_ | F_IS_ | σ^2^ | S_E_ |
| --- | --- | --- | --- | --- | --- | --- |
| aBJ | 0.1067 | 0.0389 | 0.0016 | 0.0373 | 0.0527 | 0.0102 |
| aFL | 0.0892 | 0.0812 | 0.0026 | 0.0000 | 0.0000 | 0.0000 |
| mBoc | 0.1170 | 0.0266 | 0.0013 | 0.0797 | 0.0604 | 0.0257 |
| mCJ | 0.1122 | 0.0246 | 0.0012 | 0.0797 | 0.0612 | 0.0269 |
| mDM | 0.1140 | 0.0256 | 0.0012 | 0.0751 | 0.0631 | 0.0189 |
| mFX | 0.0872 | 0.0796 | 0.0024 | 0.0000 | 0.0000 | 0.0000 |
| mI | 0.1097 | 0.0275 | 0.0013 | 0.0553 | 0.0564 | 0.0139 |
| mMV | 0.0941 | 0.0853 | 0.0025 | 0.0000 | 0.0000 | 0.0000 |
| mPC | 0.1090 | 0.0255 | 0.0012 | 0.0654 | 0.0547 | 0.0218 |
| mPD | 0.1111 | 0.0359 | 0.0015 | 0.0392 | 0.0536 | 0.0073 |
| mPG | 0.1096 | 0.0273 | 0.0013 | 0.0530 | 0.0548 | 0.0132 |
| mSB | 0.1133 | 0.0277 | 0.0013 | 0.0559 | 0.0564 | 0.0115 |

* Mean value of π in this population (π); mean measure of F_IS_ in this population (F_IS_).
